# Single-chromosome dynamics reveals locus-dependent dynamics and chromosome territory orientation

**DOI:** 10.1242/jcs.260137

**Published:** 2023-02-27

**Authors:** Yu-Chieh Chung, Madhoolika Bisht, Jenna Thuma, Li-Chun Tu

**Affiliations:** ^1^ Department of Biological Chemistry and Pharmacology, The Ohio State University, Columbus, OH 43210, USA; ^2^ Department of Molecular Genetics, The Ohio State University, Columbus, OH 43210, USA; ^3^ Interdisciplinary Biophysics Graduate Program, The Ohio State University, Columbus, OH 43210, USA; ^4^ Center for RNA Biology, The Ohio State University, Columbus, OH 43210, USA; ^5^ The Ohio State University Comprehensive Cancer Center, The Ohio State University, Columbus, OH 43210, USA

**Keywords:** Chromatin dynamics, Single-particle tracking, CRISPR, Live-cell imaging

## Abstract

Dynamic chromatin organization instantly influences DNA accessibility through modulating local macromolecular density and interactions, driving changes in transcription activities. Chromatin dynamics have been reported to be locally confined but contribute to coherent chromatin motion across the entire nucleus. However, the regulation of dynamics, nuclear orientation and compaction of subregions along a single chromosome are not well-understood. We used CRISPR-based real-time single-particle tracking and polymer models to characterize the dynamics of specific genomic loci and determine compaction levels of large human chromosomal domains. Our studies showed that chromosome compaction changed during interphase and that compactions of two arms on chromosome 19 were different. The dynamics of genomic loci were subdiffusive and dependent on chromosome regions and transcription states. Surprisingly, the correlation between locus-dependent nuclear localization and mobility was negligible. Strong tethering interactions detected at the pericentromeric region implies local condensation or associations with organelles within local nuclear microenvironments, such as chromatin–nuclear body association. Based on our findings, we propose a ‘guided radial model’ for the nuclear orientation of the long arm of chromosome 19.

## Teaser

**Summary:** Chromosome compaction depends on the cell cycle and the chromosome arm. *In vivo*, chromatin dynamics, which are modulated by transcription and interactions between chromatin and nuclear landmarks, are genomic-locus specific.

## INTRODUCTION

The human genome exists in the form of a DNA–protein complex, known as chromatin. Chromatin compaction, localization and dynamics are orchestrated for precise cellular processes. Misregulation of chromatin organization has been shown to associate with diseases including developmental defects and cancers (Akdemir et al., 2020; Lupiáñez et al., 2015). Interphase chromatin is non-randomly and hierarchically organized in the cell nucleus (Rowley and Corces, 2018). Individual chromosomes occupy discrete three-dimensional (3D) spaces with little overlaps, known as chromosome territories, a conserved feature across species. The fluorescence *in situ* hybridization (FISH) and chromosome conformation capture (3C)-based techniques have uncovered several fundamental principles of chromatin organization (Misteli, 2020). However, the diversity of genome architecture among cell types and in single cells is not fully understood. To solve dynamic genome organization problems at fine temporal scales, such as fast relaxations of chromatin domains within a few seconds, live-cell imaging approaches are necessary to provide unique insights into temporal aspects of chromatin organization changes.

Clustered regularly interspaced short palindromic repeats (CRISPR) and CRISPR-associated proteins (Cas) have been repurposed to visualize and track the movement of genomic loci in living cells (Chen et al., 2013; Ma et al., 2018, 2016b). The type II CRISPR-Cas9 system from *Streptococcus pyogenes* is one of the most commonly used CRISPR systems for genomic reorganization and imaging (Feng et al., 2020). Innately, Cas9 is an endonuclease that generates double-strand breaks on DNA. Two substitution mutations, i.e. D10A and H840A, were introduced to create the nuclease-dead version of Cas9 (dCas9) to remove the endonuclease activity (Jiang et al., 2015). Labeling telomeres by using CRISPR did not have noticeable effects on chromatin dynamics compared to the telomere movement observed after labeling the main telomeric-binding protein telomeric repeat binding factor 1 (TERF1, also known as TRF1) (Chen et al., 2013). In the multicolor CRISPR-based DNA imaging system CRISPR-Sirius (Ma et al., 2018), engineered single guide RNAs (sgRNAs) with multiplexed RNA aptamers obtained from bacteriophages PP7 and MS2 were used with its fluorescence-labeled RNA coat proteins to simultaneously track the movement of two loci on a single chromosome (Ma et al., 2018). The advantages of CRISPR-based imaging techniques are: (1) single-chromosome studies with endogenous DNA sequences; (2) precise CRISPR targeting, as CRISPR-Cas9 fails to form stable interactions with its DNA target when one or more nucleotide mismatches have been introduced between the sgRNA and the DNA-targeting site (Hsu et al., 2013; Ma et al., 2016a); (3) more than 1000 loci in the human genome can be labeled based on database search results and; (4) higher resolution can be achieved by using a shorter genomic targeting length; for example, two loci that are 4.6 kb apart can be resolved using a target size of ∼1 kb (Ma et al., 2019).

During interphase, genomic loci undergo diffusive motions within a radius of several hundred nanometers (Amitai et al., 2015; Chubb et al., 2002; Dickerson et al., 2016; Wang et al., 2008). The mobility of genomic loci depends on their location on the chromosome, transcriptional activity and interaction with nuclear landmarks, such as nuclear lamina, the nuclear pores, the pericentromeric heterochromatin and the nucleolus. We and others have shown previously that telomeres have higher mobility than loci in the interior and pericentromeric regions of a chromosome (Ma et al., 2016b; Vivante et al., 2020). However, the loci measured were not located on the same chromosome or at similar nuclear localization sites. This variation regarding the motion of genomic loci is expected when distinct local environments are present or chromatin is tethered to nuclear landmarks (Chubb et al., 2002). For example, the centromeric region of chromatin is more condensed compared with other regions of a chromosome (Gilbert and Allan, 2001). Chromatin domains in pericentromeric regions tend to cluster and have increased levels of methylated histones as compared to those in interior chromosomal regions (Déjardin, 2015). On the one hand, lamina-associated domains (LADs) and nucleolus-associating domains (NADs) are enriched within pericentromeric regions, which can promote attachment to nuclear lamina and nucleoli (van Schaik et al., 2020). On the other hand, telomeric regions are bound by highly dynamic telomerase and its cofactors (Schmidt et al., 2016). Although locus-dependent chromatin dynamics have been detected, it is still unclear whether the p- and q-arm of a single chromosome share similar locus-dependent chromatin dynamics, and chromatin compaction levels.

The position of individual chromosomes or chromatin domains relative to the nuclear center and periphery, known as ‘chromatin radiality’, has been used to classify 3D genome organization in the nucleus (Girelli et al., 2020). Peripheral nucleosomes have decreased mobility compared to that of interior nucleosomes (Shinkai et al., 2016), suggesting the existence of local chromatin condensation or attachment of peripheral chromatin to the nuclear lamina or inner nuclear membrane. However, the correlation between nuclear localization and dynamics of loci on a single chromosome remains to be elucidated. The radial position of chromosome territories has been studied using 3C-based techniques (Das et al., 2020). Although it is important to observe average positions of chromosome territories, knowing how chromosome territories are distributed across a cell population allows us to understand their stability. Yet, the orientation of chromosome territories or that of a large chromatin domain, i.e. tens of megabases, relative to the nuclear radial axis of the cell nucleus has not been systematically investigated in living cells.

Interactions between chromatin and nuclear landmarks are crucial for chromatin localization, organization, dynamics and function in the cell nucleus (Hildebrand and Dekker, 2020) as, for example, gene loci tethered to the nuclear lamina localize to the nuclear periphery and are often suppressed. However, measuring the tethering strength of loci to their surrounding nuclear landmarks is challenging in living cells. High-resolution genome-wide data measured by 3C-based techniques provide valuable information on chromatin organization via intra- and inter-chromosomal contact frequencies but, data on tethering strength – the key to mechanically understand dynamic chromatin organization – are not directly available from these studies (See et al., 2022). The dynamics, nuclear localization and tethering of genomic loci can correlate to transcription activity, and can be used to map the inhomogeneous distribution of active and inactive genes. Here, we use CRISPR-Sirius imaging to measure the biophysical properties of genomic loci on human chromosome 19, and to characterize variations and correlations in different genomic regions along a single chromosome.

## RESULTS

### Tagging and imaging genomic loci by using CRISPR-Sirius

**Fig. 1.**
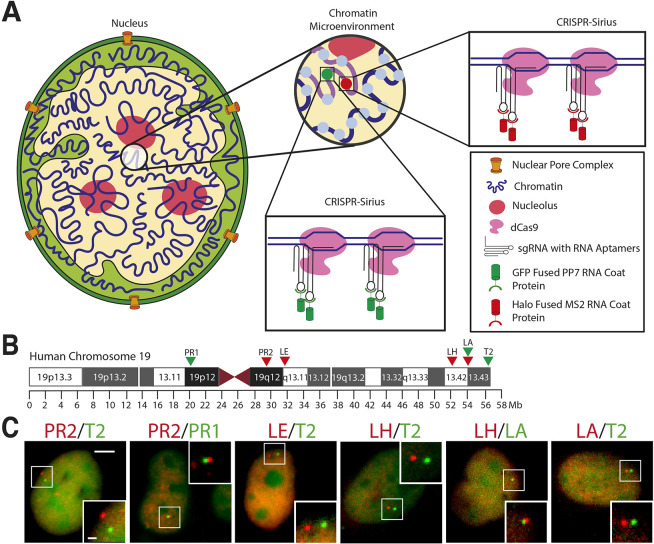
**Dual-color imaging of locus pairs by CRISPR-Sirius.** (A) Diagram of the dual-color CRISPR-Sirius system for specific genomic imaging in living cells (Ma et al., 2018). (B) G-banding ideogram of human chromosome 19, showing the location of the labeled loci PR1, PR2, LE, LH, LA and T2. (C) Visualization of dual-color-labeled genomic locus pairs in U2OS cells. Boxed areas are shown magnified in bottom right or top right corner of each image. Scale bars: 5 µm (main image); 1 µm (magnified image). Green or red (false colors) indicate loci labeled by hU6-sgRNA-Sirius-8XPP7-GFP or mU6-sgRNA-Sirius-8XMS2-halo tag-JF549, respectively.

The experimental design for labeling and imaging specific genomic loci is based on our previous study, introducing CRISPR-Sirius as an efficient system to label genomic loci on the same chromosome (Fig. 1A) (Ma et al., 2018). Two octets of RNA aptamers obtained from bacteriophages MS2 and PP7 were inserted into separate sgRNA scaffolds to generate sgRNAs for simultaneous dual-color labeling of specific genomic loci, enabling live-cell imaging with enhanced brightness. The RNA aptamers were designed and linked by three-way junctions to enhance thermostability and prevent fast degradation of sgRNAs (Ma et al., 2018), within the nucleus of human osteosarcoma U2OS cells. PP7 and MS2 coat proteins (PCP and MCP, respectively) were fused to green fluorescent protein (GFP) or the HaloTag, generating GFP-tagged PCP (PCP-GFP) and HaloTag-labeled MCP (MCP-HaloTag), and used to fluorescently label Sirius sgRNAs in live cells. The membrane permeable dye JF549 was then added to further label the HaloTag (Grimm et al., 2015). To enable imaging and obtain strong fluorescence signals, we searched human chromosome 19 for loci with a low number – i.e. between 29 and 160 – of repetitive sequences (Table 1). Six loci were selected, including a locus near the telomeric region (T2), three loci on the long arm (LA, LH, LE) and two loci in the pericentromeric regions (PR1, PR2) (Fig. 1B). As control, selected genomic loci were examined for gene activity using RNA-sequencing (RNA-seq). All selected loci were either located within intergenic regions, or within inactive genes with negligible (<0.1) or undetectable transcripts per million (TPM) (Table 1). All sgRNAs showed well-targeted pairs of dual-color-labeled loci in U2OS cells (Fig. 1C).

**Table 1.**
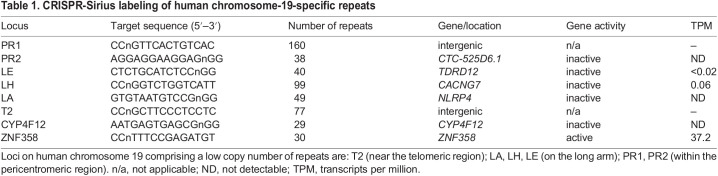
CRISPR-Sirius labeling of human chromosome-19-specific repeats

### Chromosome compaction during interphase depends on the cell cycle and the chromosome arm

**Fig. 2.**
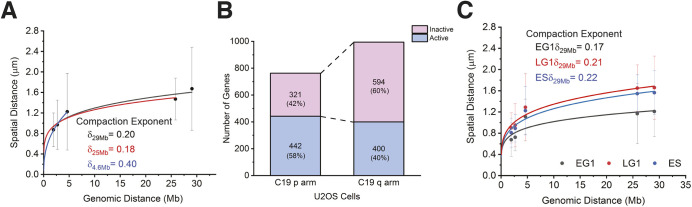
**Compaction of human chromosome 19.** (A) Mean spatial distance over the genomic distance within 4.8 Mb (blue), 25 Mb (red) and 29 Mb (black) for the q arm of chromosome 19, resulting in the compaction exponent (δ) values of the power-law relationship. The data points from left to right indicate the mean spatial distance of locus pairs LH/LA, LA/T2, LH/T2, LE/T2 and PR2/T2. Number of cells analyzed were *n*=36 (LH/LA), *n*=41 (LA/T2), *n*=41 (LH/T2), *n*=27 (LE/T2) and *n*=28 (PR2/T2). Error bars indicate the mean±s.d. (B) RNA-seq analysis of U2OS cells. Bar graphs showing number of genes actively transcribed (blue) versus inactive (pink) genes on the p and q arm of chromosome 19. (C) Chromosome 19 q arm compactions in early G1 (EG1), late G1 (LG1) and early S (ES) phase. Number of cells analyzed: *n*=25 (EG1), *n*=29 for (LG1), *n*=27 (ES). Error bars indicate the mean±s.d.

To measure the compaction of the long (q) arm of chromosome 19, we paired its genomic loci with various genomic distances, i.e. 1.93 Mb (LH/LA), 2.69 Mb (LA/T2), 4.62 Mb (LH/T2), 25.82 Mb (LE/T2), and 29.05 Mb (PR2/T2) (Table S1). The average spatial distance of these paired loci was measured and plotted against their genomic distance (Fig. 2A). The compaction level of chromatin is determined by the compaction (also known as scaling) exponent (δ) of the power–law relationship between the spatial distance and genomic distance of locus pairs (Tark-Dame et al., 2011). As shown in Fig. 2A, compaction depending on genomic length was observed on the chromosome 19 q arm, i.e. δ_4.6Mb_=0.40 for 4.6 Mb genomic size, δ_25Mb_=0.18 for 25.8 Mb genomic size, and δ_29Mb_=0.20 for 29 Mb genomic size (Table S1). Surprisingly, when comparing the compaction exponents at ∼25 Mb, we found that the q arm of chromosome 19 was packed tighter (δ_25Mb_=0.18) (Fig. 2A) than its short (p) arm (δ_21Mb_=0.22; see data published by Ma et al., 2019). Analysis of U2OS RNA-seq data indicated that the number of active genes on the p arm is ∼10% higher than those on the q arm (442 vs 400) (Fig. 2B), although the total number of genes over ∼30Mb on the q arm (*n*_q_=994 over 30 Mb) is higher compared to that on the p arm (*n*_p_=763 over 29 Mb). Of those total gene numbers on each arm, ∼40% are active on the q arm versus ∼58% on the p arm, with a higher number of active genes potentially indicating reduced nucleosome density and more active transcription factor interactions. We reasoned that a high density of active genes within a chromosomal region could lead to a more extended and relaxed configuration of chromatin.

To study how the compaction of chromosome 19 q arm changes during the cell cycle, we measured the spatial distance of locus pairs at three stages of the cell-cycle – early G1 (EG1), late G1 (LG1) and early S (ES). We found the compaction level of the q arm during EG1 to be tighter (δ_29Mb_=0.17) than that at LG1 (δ_29Mb_=0.21) and ES (δ_29Mb_=0.22), but similar during LG1 and ES (Fig. 2C). For each a locus pair, the average spatial distance increases significantly from EG1 to LG1 and remains similar from LG1 to ES, reflecting the decondensation process during transition from EG1 to LG1 (Fig. S5). These findings demonstrate the dynamic nature of chromatin organization throughout the cell cycle.

### Genomic locus dynamics are subdiffusive and locus-dependent

**Fig. 3.**
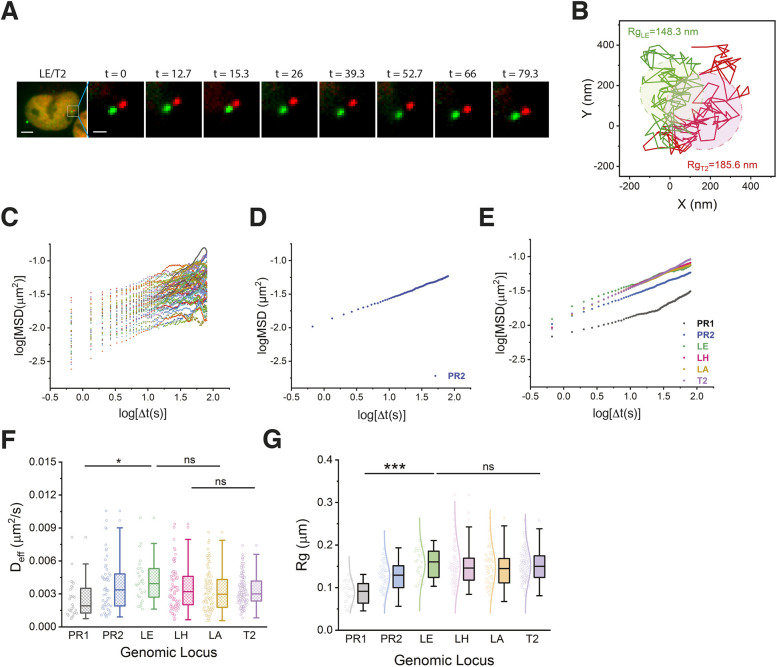
**Chromatin dynamics of human chromosome 19.** (A) Time-lapse images of the LE/T2 locus pair over 80 s (total frame number=120). The boxed area of the first image is shown magnified in all following images. Scale bars: 5 µm (first image), 1 µm (second image). (B) Trajectories of loci LE and T2 as obtained from A. Gyration radii (R_g_) of trajectories are indicated in similar colors. (C) Time-averaged MSD plot of locus PR2 at natural logarithmic scale (*n*=52 trajectories). (D) Ensemble-averaged MSD values from C. (E) Ensemble-averaged MSD curves of all six loci; *n*=28 (PR1), *n*=52 (PR2), *n*=27 (LE), *n*=77 (LH), *n*=77 (LA), *n*=127 (T2). (F) Box-and-whisker plot showing the effective diffusion constant (*D_eff_*) of all six loci. Data points are shown to the left of each box. (G) Box-and-whisker plot showing R_g_ values of individual loci (PR1, PR2, LE, LH, LA, T2). Data points and distribution curves are indicated to the left of each box. In F and G, statistical significance was assessed by one-way ANOVA; *P<0.05, ***P<0.0005 among the pericentromeric loci (PR1 and PR2) and the interior locus (LE). ns, not significant (*P*>0.05) was found among interior loci (LE, LH, LA) and the near-telomeric locus (T2).

We have previously characterized the motion of five loci – intergenic DNA regions 1, 2, 3 and 4 (IDR1, IDR2, IDR3 and IDR4, respectively), and the locus of the gene encoding transcription factor 3 (*TCF3*) – located on human chromosome 19 p arm within genomic coordinates of 0 to 4.6 Mb that only covers 7.8% of chromosome 19 (59 Mb) (Ma et al., 2019). To improve the understanding of single-chromosome dynamics, six new loci (Fig. 1B) were selected to expand loci coverage over the whole chromosome 19. Movement of a locus was recorded in 120 consecutive image frames for 80 s (Fig. 3A; Movie 1), with the trajectory of the locus given by the time series of its position on each frame (Fig. 3B). The quantification of genomic loci movement was carried out by comparing biophysical parameters – the diffusion constant and exponent of the mean square displacement (MSD) and the gyration radius (also known as trajectory radius, R_g_) of a locus trajectory (Fig. 3C–G).

We calculated the MSD from the locus trajectories. The time-averaged MSD of individual trajectories (Fig. 3C) and the ensemble-averaged MSDs of loci over cell population were calculated (Fig. 3D,E). Our data were fitted to the power-law MSD function (MSD=4*D_app_*Δt^β^), and apparent diffusion constants (*D_app_*) and diffusion exponents (β) were extracted (Table S2). The resulting power-law MSD functions indicated that locus dynamics are subdiffusive. Consistent with our previous study of genomic loci on the p arm of chromosome 19 (Ma et al., 2019), the diffusion exponents were found to be between 0.35 and 0.46 (Table S2), indicating different levels of subdiffusive motions of loci along chromosome 19. The mobility of loci can be seen from their MSD power-law curves (Fig. S1). Although MSD curves of the near-telomeric locus T2 overlap briefly with those of interior loci LA and LH, T2 mobility became less restricted after 35 s, and was the highest throughout the duration of our measurements (80 s). It is noteworthy that, at each time point, the interior loci and the locus near the telomere showed higher mobility than loci at the pericentromeric region. In less than 10 s – and for ∼30 s – the LE locus possessed highest mobility. To quantify the short-time locus dynamics, we computed the effective diffusion constants (*D_eff_*) and found locus LE had the highest average *D_eff_* value (0.00418 µm^2^ s^−1^), whereas locus PR1 at the pericentromeric region had the lowest value (0.00251 µm^2^ s^−1^). PR2, LH, LA and T2 had similar *D_eff_* values (0.00386 µm^2^ s^−1^, 0.00350 µm^2^ s^−1^, 0.00333 µm^2^ s^−1^and 0.00332 µm^2^ s^−1^, respectively) (Fig. 3F; Table S4). These results are consistent with the short-time behavior of the MSD power-law values (Table S4). Apart from using MSDs, the mobility of a locus can be characterized by its gyration radius R_g_, i.e. the area covered by its trajectory within a given time, and can be regarded as ‘locus territory’. We found similar average gyration radii (Fig. 3G) for interior loci, i.e. 1.46×10^−1^ µm (LA), 1.51×10^−1^ µm (LH), 1.54×10^−1^ µm (LE), and 1.52×10^−1^ µm (telomeric locus T2). However, the gyration radii of loci at pericentromeric regions PR1 and PR2 were significantly smaller with 0.88×10^−1^ µm and 1.28×10^−1^ µm, respectively (Table S2). This suggests that the movement of loci within the pericentromeric regions was more constrained than that of loci within the interior and telomeric regions for both arms of chromosome 19. To exclude the effects from transcription activities, we analyzed genomic loci that are either located within intergenic regions or within genes that are not transcribed in U2OS cells (Table 1). The different mobility among genomic loci within a short-time period (<1 s) was mainly caused by the variability of chromatin–chromatin interactions, i.e. inter-locus interaction, and chromatin–environment attachments, such as those of nuclear organelles.

### Transcriptional inhibition increases the dynamics of a transcriptionally active locus but not that of a silenced gene

**Fig. 4.**
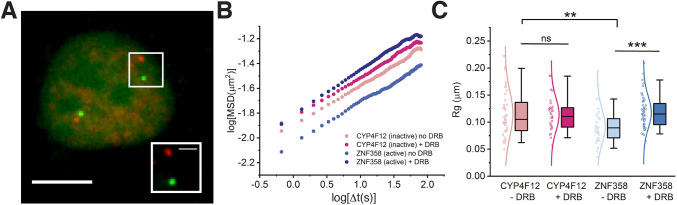
**Effects of transcriptional activities on chromatin dynamics.** (A) Image showing the genomic *CYP4F12* and *ZNF358* loci in a U2OS cell. Green and red false colors indicate labeling with hU6-ZNF358-Sirius-8XPP7-GFP and mU6-CYP4F12-Sirius-8XMS2-halo tag-JF549, respectively. The boxed area is shown magnified in the bottom right corner. Scale bars: 5 µm (main image); 1 µm (magnified image). (B) Ensemble-averaged mean square displacement (MSD) values of silenced CYP4F12 treated (red) or not treated (pink) and the non-silenced ZNF358 locus treated (dark blue) or not treated (light blue) with DRB to inhibit transcription. (C) Box-and-whisker plot of gyration radii (R_g_) of individual loci with (+) and without (−) DRB treatment. Data points and distribution curves are indicated to the left of each box. Statistical significance was assessed by two-tailed Welch's *t*-test (95% confidence level), ***P<0.0005 for non-silenced ZNF358 (+DRB and −DRB); **P<0.005 for silenced CYP4F12 (−DRB) and non-silenced ZNF358 (−/+DRB). ns, not significant (*P*>0.05) for silenced CYP4F12 (+DRB or −DRB). Trajectories: CYP4F12 −DRB (*n*=40), CYP4F12 +DRB (*n*=31), ZNF358 −DRB (*n*=41), ZNF358 +DRB (*n*=56).

How transcription activities affect genomic locus movement is not fully understood. Nozaki et al. reported increased nucleosome movements when the cells were treated with the RNA polymerase II elongation inhibitor 5,6-dichloro-1-β-D-ribofuranosylbenzimidazole (DRB), a (Nozaki et al., 2017). However, the effects of transcription inhibition on chromatin mobility at active versus inactive genes remain unknown. To investigate transcription effects on chromatin dynamics, two genomic loci on the p arm of chromosome 19 were chosen on the basis of gene activities, one locus within non-silenced *ZNF358* (hereafter referred to non-silenced ZNF358 locus) (TPM=37.2) and another locus within the silenced *CYP4F12* (hereafter referred to as silenced CYP4F12 locus) as a control (TPM=0) (Table 1). We used DRB to block transcription and tracked the mobility of loci with and without DRB treatment (Fig. 4; Table S3). Without DRB treatment, the silenced CYP4F12 locus showed higher mobility than that of the non-silenced ZNF358 locus. Mobility of the non-silenced ZNF358 locus increased upon transcription inhibition by DRB, whereas mobility of the silenced control CYP4F12 locus remained the same. These findings indicate the negative effects of the transcription machinery on chromatin dynamics, i.e. transcription promotes chromatin rigidity at actively transcribed regions, contributing to the heterogeneous organization of chromatin within a single chromosome.

### Distribution of genomic loci along the nuclear radial axis and preferred nuclear locations for pericentromeric and telomeric regions

**Fig. 5.**
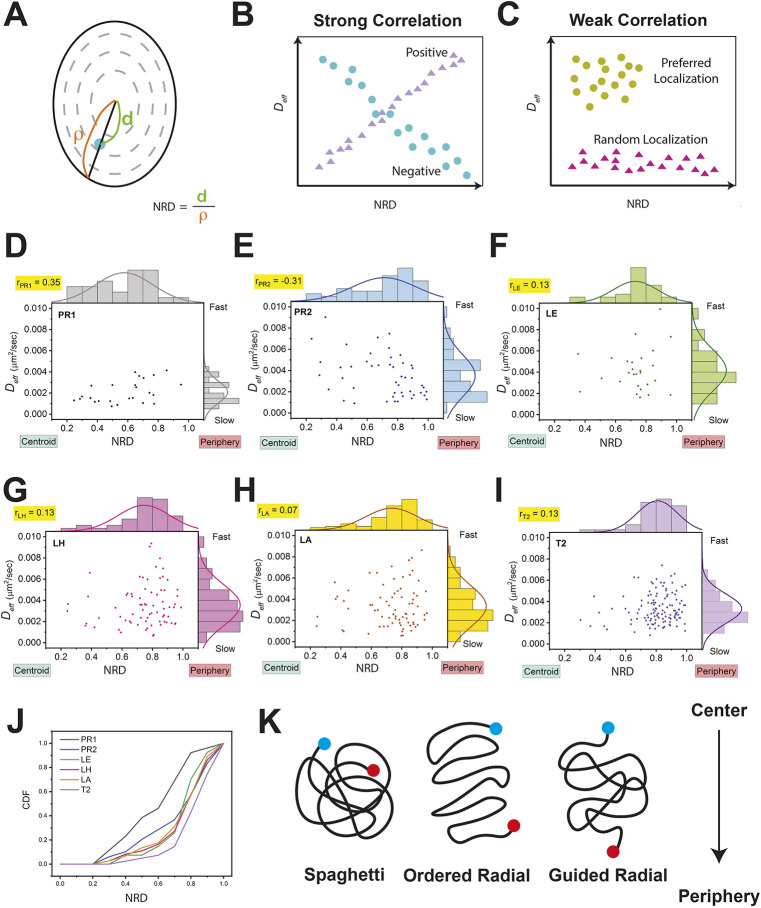
**Correlation analysis between nuclear radial distances and effective diffusion constants.** (A) The normalized nuclear radial distance (NRD) is defined by the ratio of the distance from the nuclear centroid to the genomic locus (d) over the distance from the nuclear centroid to the nuclear boundary (ρ). (B) Diagram showing positive and negative correlation between the radial distance and effective diffusion constant (*D_eff_*). (C) Diagram of the weak correlation between the radial distance and *D_eff_* with and without a preferred distribution of location. (D–I) Scatter plots of nuclear radial distance and *D_eff_* values of individual genomic loci as indicated within each plot. The top-left corner of each panel states the Pearson's correlation coefficient (r). Histograms on the top and right side of each panel indicate the distribution of locus-normalized radial distances and *D_eff_* values, respectively. (J) Plotted is the cumulative distribution function (CDF) of all genomic locus-normalized radial distances, with PR1 in gray, PR2 in blue, LE in green, LH in pink, LA in yellow and T2 in violet. (K) Models (spaghetti, ordered radial and guided radial) of chromosome 19 long arm orientation and locus localization in the cell nucleus. Dots represent genomic loci at centromeric (blue) and telomeric (red) regions. The nuclear–radial direction, i.e. the direction from the nuclear centroid to the nuclear boundary, is indicated by an arrow.

To investigate whether the dynamics of genomic loci depend on their nuclear localization, we plotted the *D_eff_* values of loci against their normalized radial distance (NRD) (Fig. 5A). We determined strong correlations when linear distributions with clear upward or downward trends were observed (Fig. 5B). However, a flat or random distribution of a dataset indicates weak or no correlations (Fig. 5C). The correlation can be measured and classified by correlation coefficients. A preferred localization was found when data points accumulate at a specific nuclear radial distance.

Consistent with data from fluorescence *in situ* hybridization (FISH) experiments in fixed cells (Bolzer et al., 2005), we found that specific genomic loci on chromosome 19 have a wide range of nuclear radiality (Fig. 5D–I, top histogram) – although chromosome 19 has been reported as an interior chromosome. Surprisingly, genomic loci on different regions of chromosome 19 favored distinct nuclear radial distributions. PR1 on the p arm of chromosome 19 tended to localize towards the nuclear center with an almost even radial distribution (NRD=0.2–0.8). Another pericentromeric locus, PR2, closest to PR1 but in the q arm, was able to localize to the nuclear center or periphery (with NRD≤0.8). Loci in interior regions of the q arm, i.e. LE, LH and LA, showed values of NRD=0.7–0.8. More peripheral localizations were found on the near-telomeric locus T2 with NRD≤0.8–0.9. Cumulative distribution functions (CDFs) also confirmed the preferred NRD value for each locus (Fig. 5J).

### Genomic locus dynamics have little or no correlation to the nuclear radial distance

To investigate the correlation of locus mobility versus nuclear localization, we calculated the Pearson's correlation coefficients (r) for the scatter plot of nuclear radial distances and *D_eff_* values for all loci (Fig. 5D–I, the number on the top-left corner). Interestingly, none of the loci showed a significant correlation between the nuclear radial distance and *D_eff_* values, suggesting that the mobility of a locus cannot be simply classified by its nuclear localization. Negligible Pearson's correlation coefficients (r<0.2) were found for loci of interior (LE, LH, LA) and near-telomeric regions (T2), but weak correlations were found for loci at pericentromeric regions (PR1, PR2). Surprisingly, PR1 on the p arm has a weak positive Pearson's correlation coefficient (r_PR1_=0.35), suggesting a mild elevation of *D_eff_* values for loci located near the nuclear periphery. In contrast to PR1, the PR2 locus on the q arm showed a weak negative Pearson's correlation coefficient (r_PR2_=−0.31), meaning the mobility of the PR2 locus reduced when localized to the nuclear periphery. Decreased mobility was primarily observed when PR2 localized to the nuclear periphery with an NRD of 0.8–1.0 but not in regions with values of NRD<0.8. This result suggests nuclear radial localization had no influence on locus mobility on chromosome 19, except for the weak Pearson's correlation coefficients at pericentromeric regions.

We also noticed that all six loci have distinct ranges of *D_eff_* distributions (Fig. 5D–I, right histogram), with the *D_eff_* distribution for PR1 being the narrowest. On the q arm, the *D_eff_* distribution of PR2 and T2 was narrower compared to that of other loci; the widest range was found for loci located closer to the interior region of the chromosome. When the locus is less constrained, the range of *D_eff_* distribution was wider. Short-time locus dynamics are mainly subject to local interactions, whereas the crosslinking of chromatin may contribute to the diffusion exponent of subdiffusive long-term locus dynamics (Amitai et al., 2017). Thus, the range of *D_eff_* distribution might reflect the frequency of interactions between the locus and its local environment. Strong local interactions that constrain locus motions lead to reduced *D_eff_* values and a narrow *D_eff_* distribution of the loci. As a result, narrow *D_eff_* distributions on loci within pericentromeric and near-telomeric regions suggest a high frequency of locus-local environment interactions, such as interactions between loci and nuclear landmarks.

### The ‘guided radial’ model

Based on our nuclear radial and *D_eff_* distributions of loci, and averaged spatial distance of locus pairs on chromosome 19, we propose a ‘guided radial’ model (Fig. 5K). In this model, chromosome territories have preferred orientations guided, perhaps, by interactions between specific chromosomal regions and nuclear landmarks. In the case of human chromosome 19, all genomic loci on the interior q arm consistently showed a preferred radial distance of ∼0.8. On the one hand, loci in the pericentromeric regions were mainly located in the central region with NRD values mostly below 0.8. On the other hand, the near-telomeric locus T2 is primarily located in the periphery, at an NRD of 0.8∼1.0. Two extreme variants of the guided radial model are ‘spaghetti’ and ‘ordered radial’ models (Fig. 5K). In the spaghetti model, a random organization with no tendency of nuclear radial distribution on any genomic loci should be observed. In the ordered radial model, a gradient preference of nuclear radial distributions according to their genomic locations along the chromosome and some level of rigidity to maintain the ordered structure are expected. Neither the spaghetti nor the ordered radial model was observed in our data analysis.

### Chromatin elasticity and tethering to its local environment

**Fig. 6.**
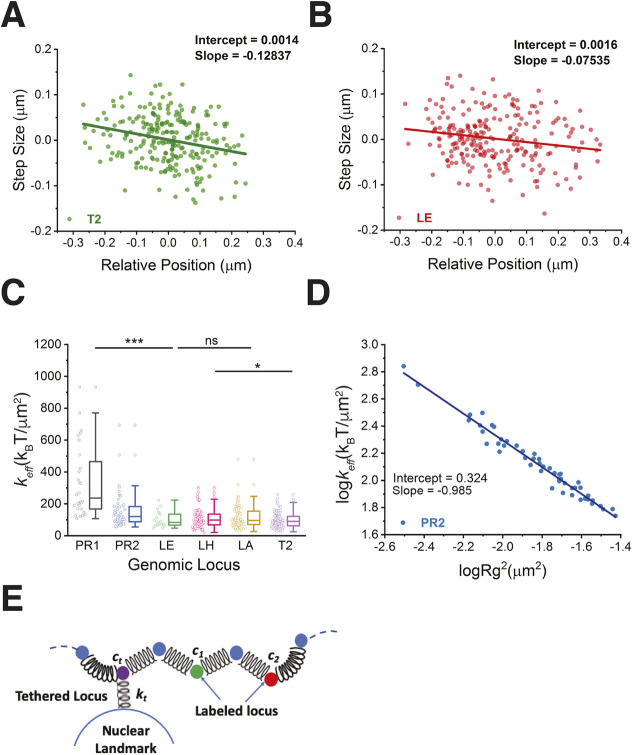
**Dynamics and tethering of loci along human chromosome 19.** (A,B) Scatter plots of the step size of locus movement between two consecutive time points versus the relative position to the centroid of locus trajectory for loci T2 (A) and LE (B) as shown in Fig. 3B. Data were fitted by linear regression (solid line). (C) Box-and-whisker plot of effective spring constants (*k_eff_*); trajectories: PR1 (*n*=28), PR2 (*n*=52), LE (*n*=27), LH (*n*=77), LA (*n*=77), T2 (*n*=127). Data points are indicated to the left of each box. (D) Scatter plot of the effective *k_eff_* values versus the locus territory of PR2 (R_g_^2^) at log/log scale. Data were fitted with a linear fit (solid line). (E) Schematic of a Rouse polymer chain with a tethered locus (c_t_, purple circle), two labeled loci (c_1_ and c_2_, green and red circles) and the tethering spring constant (k_t_). Blue circles indicate nearby loci without fluorescence labeling. Statistical significance was assessed by one-way ANOVA (*P<0.05; ***P<0.0005; ns, not statistically significant, *P*>0.05).

We showed here that the dynamics of loci on chromosome 19 are subdiffusive. The subdiffusive dynamics of genomic loci can be modeled by the generalized Langevin equation (Lampo et al., 2016; Lucas et al., 2014). External forces that constrain the mobility of a genomic locus include inter–locus interactions, the tethering interaction of a locus to the local environment and the frictional force from its surrounding medium. For short time periods, the system can be modeled by the normal Langevin equation (Vivante et al., 2020) in which the locus motion can be approximated to a normal diffusion with *D_eff_*, and the local nucleoplasm can be treated as a viscous medium with a friction coefficient γ related to *D_eff_* by the Einstein relation γ=*k*_B_*T/D_eff_*, where *k*_B_ is the Boltzmann constant and T the absolute temperature of the environment. In this model, the effective external force applied on the locus is assumed to obey Hooke's law with an effective spring constant *k_eff_* (Amitai et al., 2015). The *k_eff_*  value, calculated from the linear regression of the step size of locus movement between two consecutive time points versus the relative position of the locus to the centroid of the trajectory, can be used to measure the strength of local effective forces or interactions applied on the loci (Vivante et al., 2020) (Fig. 6A,B). We found that the *k_eff_* of the locus at the pericentromeric region (∼154 *k*_B_T µm^−2^) is significantly higher than those of loci at the interior (∼110 *k*_B_T µm^−2^) and near-telomeric regions (∼97 *k*_B_T µm^−2^) on the chromosome 19 q arm. Similarly, we found that the locus PR1 (pericentromeric region) on chromosome 19 p arm possesses a high *k_eff_* (∼336 *k*_B_T µm^−2^) (Fig. 6C; Table S4). The *k_eff_* of the locus is expected to inversely correlate with the locus territory (Figs 6C, 3G). Polymer model predicts the relationship <*k_eff_*>=a<*R_g_^2^*>^−b^, where a=2 and b=1 (Amitai et al., 2015). Our data showed that (a_PR1_, a_PR2_, a_LE_, a_LH_, a_LA_, a_T2_)=(2.09, 2.11, 2.48, 2.44, 2.65, 2.44) and (b_PR1_, b_PR2_, b_LE_, b_LH_, b_LA_, b_T2_)=(0.99, 0.99, 0.94, 0.95, 0.93, 0.94), which is consistent with the theoretical prediction (Fig. 6D; Fig. S2).

To further extract the information regarding inter–locus interactions and tethering interactions between a locus and its environment, we modeled the chromosome by using the Rouse polymer model (Doi and Edwards). For simplicity, we considered the polymer chain with one tethered locus at position c_t_, and two labeled loci at positions c_1_ and c_2_ (Fig. 6E). In this model, *k_eff,i_=k_t_* K*_R_/*(K*_R_+*Δc_i*t*_ *k_t_*), where K*_R_* is the *k_eff_* of the polymer chain, *k_t_* is the tethering spring constant, and Δc_i*t*_ is the distance between c_t_ and c_i_ (Amitai et al., 2015). For the locus pair LA/T2, we obtained the average effective spring constant K*_R_*∼25.0 (*k*_B_T µm^−2^) and *k_t_*∼59.3 (*k*_B_T µm^−2^) (Supplementary Information). For the tethering spring constant, measuring the strength of locus tethering near the pericentromeric region, we obtained *k_t_*∼193.5 (*k*_B_T µm^−2^) and 609.7 (*k*_B_T µm^−2^) at tethered loci near PR2 and PR1, respectively, by assuming the same value of *K_R_* and Δc_i*t*_=1 Mb. The simulation of the β-polymer model (Amitai and Holcman, 2013) showed that the crosslinking interaction among loci contributes to the *k_eff_*, which is inversely proportional to Δc_i*t*_ (Amitai et al., 2015). Because the genomic distance of the a locus pair LA/T2 is considerably smaller than the whole chromosome 19, the long-range interaction within a polymer chain can be neglected for our calculation of effective tethering spring constants. Our results suggest that the tethering interactions of loci in the centromeric region are stronger than those in the near-telomeric region.

## DISCUSSION

**Fig. 7.**
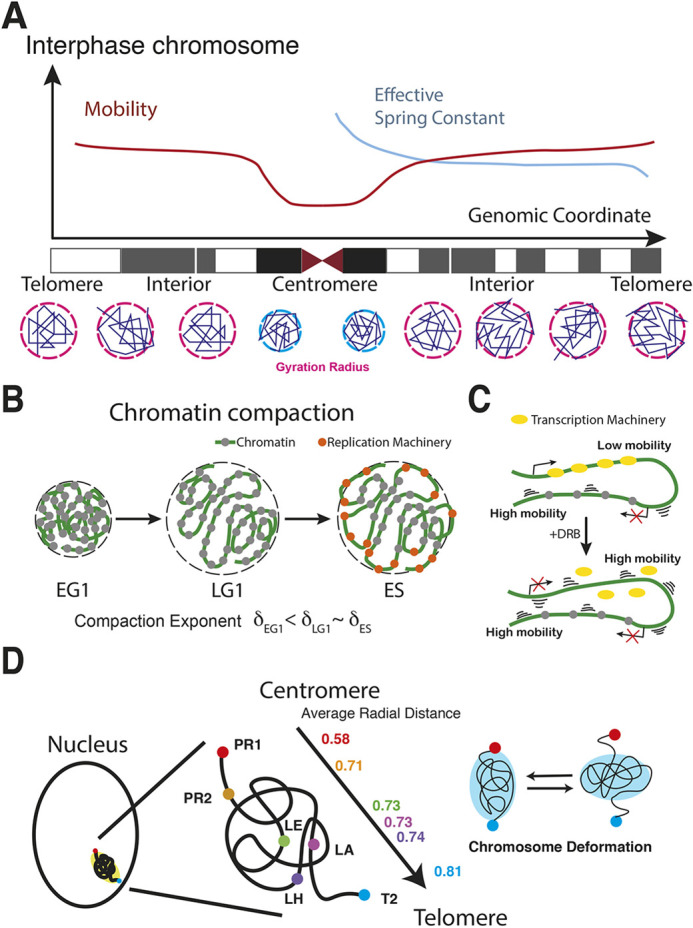
**Summary of single-chromosome dynamics, compaction and nuclear orientation.** (A) Variation of mobility and effective spring constants of genomic loci along the chromosome coordinate. (B) Chromatin compaction. Compaction exponents vary at different phases of the cell cycle. (C) Chromatin mobility reduces at transcriptionally active regions and increases upon transcription inhibition. (D) The guided radial model. Orientation of the chromosome 19 long arm favors an interior location for the centromere and a peripheral location for the telomere, shown by the radial distance of genomic loci along chromosome 19. Although our data suggest the existence of dynamic chromosome deformation, more labeling sites are required to characterize the chromosome deformation of the entire q arm.

By using CRISPR-Sirius real-time locus tracking, we characterized chromatin dynamics and compaction along a single chromosome on endogenous DNA sequence (Fig. 7A,B). The nuclear arrangement of individual chromosomes in interphase nuclei is confined to discrete 3D spaces, known as chromosome territories (Cremer and Cremer, 2001). How chromosomes are organized within the territories has been a long-standing question in the field. Chromosome conformation capture-based techniques and advanced FISH analyses have shed light on snapshots of chromosome organization in high resolution. Transcription activities and epigenetic marks have been demonstrated to have a crucial role in chromatin compaction (Bannister and Kouzarides, 2011; Nagashima et al., 2019). Compaction exponents have been used to quantitatively compare the compaction levels among chromosomal domains in different epigenetic states, such as polycomb-repressed domains (Boettiger et al., 2016), during X-chromosome inactivation (Wang et al., 2016) and lamina-induced chromosomal stretching (Sawh et al., 2020). In this work, we found that chromosome 19 p arm compaction was slightly looser than the compaction of the q arm. RNA seq analysis indicated ∼10% more active genes on the p arm of chromosome 19 than on the q arm. Our results aligned with previous FISH studies, in which actively transcribed chromosomal domains have larger compaction exponents than the compaction exponents of repressed chromosomal domains (Boettiger et al., 2016; Wang et al., 2016; Sawh et al., 2020). The plateaued compaction curves of chromosome 19 further suggested that both arms formed collapsed chromosome conformations. Although chromosome condensation during M phase has been studied, it is unknown whether decondensation is completed before entering G1 phase and whether chromosome compaction remains unchanged during interphase. Recent work by Abramo et al. demonstrated that human cells spent hours establishing topologically associating domains (TADs) and formed compartments after cells exited M phase, indicating that genome organization in early and late G1 phase are different (Abramo et al., 2019). We showed here that the compaction of chromosome 19 q arm during early G1 phase is elevated compared to that during late G1 phase, which implies that genome reorganization after mitosis is not completed in the first 3 h of G1 phase (Fig. 7B). In addition, chromosome compactions were similar between late G1 and early S phase. To further understand how cell-to-cell variability and temporal variability of spatial distance of locus pairs contributed to chromosome compaction changes during the cell cycle, we analyzed these two variations separately by using locus pairs (Figs S5,S6). Our data showed that locus pairs of shorter genomic distance, i.e. LH/LA (1.9 Mb) and LA/T2 (2.7 Mb), have cell-to-cell variations of ∼0.23–0.33 µm during all stages of the cell cycle. For locus pairs of longer genomic distance, i.e. LH/T2 (4.6 Mb), LE/T2 (25 Mb) and LD/T2 (29 Mb), the cell-to-cell variation of spatial distance was ∼0.29–0.52 µm during all stages of the cell cycle. The smaller variations at shorter genomic distances (<3 Mb) suggest restricted freedom of movement for loci within a substructure, such as a TAD. For locus pairs with a genomic distance >3 Mb and that are less likely to reside in one substructure, movement is more flexible and independent. By analyzing the temporal variation (from the time average) of spatial distance for each a locus pair, we found that the temporal variations are similar (∼0.1 µm) during all cell cycle stages (Fig. S6) and much less compared with cell-to-cell variation. Thus, the observed variation of spatial distance of locus pairs over cell cycle stages (Fig. 2C) mainly due to cell-to-cell variations. These results emphasize the importance of genome research in single living cells, in which cell-to-cell variation and temporal variation of genome organization can be examined separately. Our results and recent research of cell cycle-dependent chromosome compactions challenge the long-standing hypothesis of unchanged chromosome organization throughout the entire interphase.

The movement of genomic loci is confined and subdiffusive. However, speed, directionality and radius of locus movement depend on transcription activity, compaction of the chromatin and interactions between chromatin and nuclear landmarks (Bronshtein et al., 2015; Nagashima et al., 2019). How transcription affects the mobility of genomic loci has been controversial (Mearini and Fackelmayer, 2006; Nagashima et al., 2019). To systematically probe how transcription effects locus mobility, we analyzed genomic loci with or without active transcriptional activities, i.e. ZNF358 or CYP4F12, respectively, and measured locus mobility with or without inhibition of transcription with DRB. Our results indicated that mobility of the non-silenced ZNF358 locus comprising a moderate TPM (TPM=37.2) is significantly below that of the silenced CYP4F12 locus, suggesting negative effects of the transcription machinery on genomic locus mobility. Interestingly, the slow mobility of non-silenced ZNF358 increased upon inhibition of transcription (Fig. 7C), whereas no significant differences in mobility were found for the silenced CYP4F12 locus when transcription was inhibited or not. With the precise labeling method, our data provide unique insights into the transcription effects on chromatin dynamics locally (as a single gene) and globally (induced by the transcription inhibitor). Our results are, therefore, more detailed than the average effects of transcription on nucleosome movement across the nucleus previously reported by other groups. Our results also explain possible reasons for the controversial results regarding chromatin dynamics.

By targeting specific genomic loci in different chromosomal regions, we quantitatively determined the effects of chromosomal regions and nuclear location on the mobility of genomic loci. To test whether locus dynamics are a function of the locus position, we evaluated Pearson's correlation coefficients between the *D_eff_* value and nuclear radial position of the genomic loci. We found that the correlations between mobility and nuclear location are negligible. Notably, studies on telomeres in four cell types – mouse embryonic fibroblasts (MEFs), 3T3, HeLa and U2OS cells – also showed no significant difference in the range and degree of movement between interior telomeres and telomeres near the nuclear envelope (Vivante et al., 2017). Our initial findings prompt future research on whether nuclear localization and mobility of genomic loci are dependent on the DNA sequence (e.g. lamina-associated domain) or on the nuclear environment. Chromatin dynamics data from other chromosomes and from other cell types are necessary to determine whether our discoveries generally apply to all chromosomes.

To better understand how local interactions constrain the locus dynamics, we used the Langevin equation with an effective local force obeying Hooke's law of *k_eff_* to model short-time locus dynamics. Our results showed that the *k_eff_* values of genomic loci vary along the chromosome. Loci at the pericentromeric region have the highest *k_eff_* values; in the interior region, values then decrease until they reach a plateau then decrease further for the near-telomeric region. We measured a spring constant of T2 (the locus near the telomere) of 97 *k*_B_T/µm^2^, with its value falling in MEF cells to within the spring constant peak range of telomeres, i.e. between 75 and 100 *k*_B_T/µm^2^ (Vivante et al., 2020). The variation of *k_eff_* values was found to be inversely correlated to that of the *D_eff_* value. By calculating the power-law decay of the *k_eff_* versus locus territory and comparing it with polymer model prediction, we concluded that the short-time locus dynamics are constrained by the effective local force. To further decipher the effective force applied on loci, we modeled the chromatin elasticity and tethering interactions between loci and nuclear landmarks by using the Rouse model. The results showed that tethering interactions of loci in the centromeric region were stronger than those of loci in the telomeric region. Additionally, we found that loci with strong tethering couplings often associated with nucleoli in living cells.

In summary, we have shown that biophysical parameters, such as diffusion constants and locus territories, can provide information about the location, dynamics and interactions of a locus with local environments. The distribution of these parameters reflects the dynamic nature of chromatin. Integration of this information led to the guided radial model of the chromosome 19 q arm (Fig. 7D). In contrast to averaged genome-wide chromatin dynamics within a single nucleus, our measurements of locus dynamics at the single-chromosome level provided refined information about local interactions and dynamics of chromatin on endogenous DNA sequences. In future studies, we aim to elucidate the chromosome-specific dynamics in different cell types. Combined with polymer models, CRISPR-Sirius opens a new avenue in understanding the interactions between chromatin and nuclear landmarks, such as nuclear bodies or nuclear lamina, and interactions between gene regulatory elements that reside on chromosomal DNA, such as enhancer–promoter interactions.

## MATERIALS AND METHODS

### Plasmid construction

The sgRNA sequences and genomic coordinates are listed in Table 1 and Table S5. sgRNAs were obtained from Integrated DNA Technologies or Sigma-Aldrich and inserted into the sgRNA vectors via BbsI restriction sites. The *S. pyogenes* expression vector pHAGE-TO-dCas9-P2A-HSA,for dCas9 (nuclease-dead) was from our previous work (Ma et al., 2018). PCP-GFP and pHAGE-EFS-MCP-HaloTag have been previously described (Ma et al., 2018, 2016b). Expression vectors for dual guide RNAs, pPUR-P2A-BFP-mU6-sgRNA-Sirius-8XMS2 and pPUR-P2A-BFP-hU6-sgRNA-Sirius-8XPP7 are based on the pLKO.1 lentiviral expression system and have been described previously (Ma et al., 2018). All dCas9 and guide RNA expression vectors mentioned here are available on Addgene (Addgene plasmids 121936, 121937, 121938 and 121944).

### Cell culture, lentivirus transduction and transcription inhibition

Human osteosarcoma U2OS cells (ATCC) were cultured on 35 mm glass-bottom dishes at 37°C in Dulbecco-modified Eagle's Minimum Essential Medium (DMEM) containing high glucose and supplemented with 10% (vol/vol) fetal bovine serum (FBS). We used the U2OS^dCas9-HSA/PCP-GFP/MCP-HaloTag^ cell line that had been generated previously (Ma et al., 2019). Lentiviral particles that carry sgRNA plasmids were generated using HEK293T cells by using a previously described protocol (Ma et al., 2019). HEK293T cells were maintained in Iscove's Modified Dulbecco's Medium containing high glucose and supplemented with 1% GlutaMAX, 10% FBS and 1% penicillin/ streptomycin. At 24 h before transfection, ∼5×10^5^ cells were seeded in six-well plates. For each well, 0.5 µg of pCMV-dR8.2 dvpr (Addgene #8455), 0.3 µg of pCMV-VSV-G (Addgene #8454) – each constructed to carry HIV LTRs – and 1.5 µg of plasmid containing the gene of interest were co-transfected using TransIT transfection reagent (Mirus) according to manufacturer's instructions. After 48 h, the virus was collected by filtration through a 0.45 µm polyvinylidene fluoride filter and immediately used or stored at −80°C. For lentiviral transduction, U2OS cells were transduced in six-well plates with lentiviral supernatant for 48 h using spinfection; ∼2×10^5^ cells were then combined with 1 ml lentiviral supernatant and centrifuged for 30 min at 1200 ***g*** by. Cells were tested for mycoplasma contaminations using the MycoAlert PLUS Kit (Lonza). To inhibit transcription, 50 µg/ml polymerase II elongation inhibitor 5,6-dichloro-1-β-D-ribofuranosylbenzimidazole (DRB; Sigma-Aldrich) was added the culture medium for 3 h before imaging (Nozaki et al., 2017).

### Cell cycle synchronization

#### Cells imaged in S phase

To synchronize cells to S phase, U2OS cells were arrested during a double thymidine block (Karanam et al., 2012). For this, ∼0.5×10^5^ cells were seeded into a 35-mm imaging dish (MatTek) with 1 ml culture medium, blocked with 2 mM thymidine (VWR) for 17 h, released by rising in PBS. After 12 h, the cells were blocked again by a second exposure of thymidine. After 17 h, cells were released and images captured immediately, as early S phase.

#### Cells imaged in G1 phase

Approximately 3×10^5^ U2OS cells were seeded into a 25 cm^2^ flask containing culture medium. After 6.5 h, thymidine was added to a final concentration of 2 mM and cells were incubated at 37°C for 17 h. The cells were then washed 3× with PBS, released for 12 h and blocked again by thymidine as in the first thymidine block. After 17 h, cells were washed again and medium was replenished with 50 ng/ml nocodazole (Sigma) to induce cell cycle arrest at prometaphase (Vassilev et al., 2006). After 12 h, the cells were synchronized in M phase, harvested by shake-off, transferred to a 15 ml conical tube and collected by centrifugation. The cells were then released and seeded into a 35-mm imaging dish with fresh culture medium. Images were captured after 2 h for early G1 phase and after 7 h for late G1 phase.

### Analysis of RNA-sequencing data

Raw reads of RNA-sequencing (RNA-seq) data of human U2OS cells were obtained from Sequence Research Archive (GEO accession no. GSE118488, SRA – SRX4549306 and SRX4549307). Quality of raw data was assessed using FastQC (v. 0.72; https://www.bioinformatics.babraham.ac.uk/projects/fastqc/). Raw data were aligned to the human genome build GRCh38 using the bioinformatic tool HISAT2 (v. 2.1.0) (Kim et al., 2015), which generated the Binary Alignment Map (BAM). Reads from the BAM files were then counted using FeatureCounts (v. 1.6.4) (Liao et al., 2014). Raw read counts and read length corresponding to each gene were used to generate transcripts per million (TPM) values (Wagner et al., 2012). To identify active genes, we used the DAFS algorithm (George and Chang, 2014) based on Kolmogorov Smirnov distance statistics (Massey, 1951) to calculate the TPM cut-off for active genes in the RNA seq data set. The DAFS algorithm is based on model-based clustering and uses the R package mclust (Scrucca et al., 2016) and earth (https://CRAN.R-project.org/package=earth), to predict the cut-off value corresponding to active genes. After identifying active genes, we used the R package TxDb.Hsapiens.UCSC.hg38.knownGene (R package version 3.15.0 from Bioconductor; https://doi.org/doi:10.18129/B9.bioc.TxDb.Hsapiens.UCSC.hg38.knownGene) to obtain the genomic coordinates which correspond to each active gene on chromosome 19. The genomic coordinates were finally used to determine the number of genes on the short (p) arm and long (q) arm of chromosome 19.

### Fluorescence microscopy

We used an Olympus IX83 microscope equipped with three EMCCD cameras (Andor iXon 897), four lasers (405 nm, 488 nm, 561 nm, 647 nm), mounted with a 1.6× magnification adapter and 60× apochromatic oil objective lens (NA 1.5, coverslip- and temperature-corrected), resulting in a total of 96× magnification. The microscope stage incubation chamber was maintained at 37°C with CO_2_ and humidity supplement. A laser quad-band filter set for TIRF (emission filters at 445/58, 525/50, 595/44, 706/95) was used to simultaneously collect fluorescence signals. Imaging data were acquired by CellSens software. The localization precision was measured by capturing 120 frames of 0.1 µm coverglass-immobilized TetraSpeck fluorescent microspheres (*n*=220 for 16 s; *n*=218 for 80 s) for an exposure time of 100 ms, a method developed by Jeff Gelles (Gelles et al., 1988). The mean±s.d. from repetitive measurements of bead locations was used to represent the localization precision of our optical system. The lateral localization precision determined by 100 nm coverslip-absorbed Tetraspeck beads was ∼6 nm in 16 s (Fig. S3) and ∼10 nm in 80 s (Fig. S4). Localization precisions were slightly larger (∼1 nm difference) in the red (561 nm excitation) channel compared to the green channel (488 nm excitation) in 16 s but this difference became insignificant when total imaging time is much shorter (4 s) (Fig. S3A) or longer (80 s) (Fig. S4). When separating *x-* and *y*-axis, we observed a slightly better (∼1 nm difference) localization precision along the *y*-axis (Fig. S3B). Localization uncertainty for moving loci was 50±5.8 nm, which was estimated by fitting the MSD with a constant offset. (Renner et al., 2017). Image size was adjusted to show individual nuclei; intensity thresholds were set on the basis of the ratio between nuclear focus signals to background nucleoplasmic fluorescence. To quantify the spatial distance or track the dynamics, only locus pairs within the same focal plane were analyzed. To minimize cell cycle effects, we excluded data obtained for smaller nuclei (cells at early G1 stage) and cells with four or more foci (cells at late S, G2 and M stage, when DNA replication is completed). A final concentration of 2 nM JF549-HaloTag ligand (Promega) was added to the culture medium 12 h before imaging.

### Imaging processing

Images were registered and analyzed using *Fiji* (Schindelin et al., 2012) and *Mathematica* (Wolfram) software. Images obtained by using green and red channels were registered by 0.1 µm coverglass-absorbed TetraSpeck fluorescent microspheres (Invitrogen) as a standard sample. To eliminate movement from live cells, the localization of individual genomic loci was calibrated by the motion relative to the nuclear centroid.

### Analysis of chromosome compaction, dynamics and tethering interaction

The mean square displacement (MSD) of lag time *kΔt* was calculated by (Qian et al., 1991):

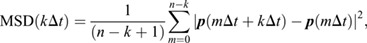
where ***p***(*t*) is the position vector of a locus at time *t*, and *Δt* is a fixed time interval between two successive image frames. All MSD curves were fitted using the power-law equation MSD(*t*)=4*D_app_ t*^β^, where *D_app_* is the apparent diffusion constant. The gyration (or trajectory) radius R*_g_* of the locus trajectory was calculated as:

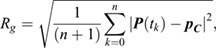
in which 
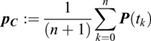
 is the geometric center of the positions defining the trajectory and *t_k+1_*=*t_k_+Δt*. The gyration radius measures the size of the area covered by locus movement and can be regarded as the territory of locus dynamics (locus territory). Chromatin compaction was measured by the compaction exponent δ of the power-law relationship, 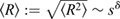, where *R* and *s* are the spatial distance and genomic distance of locus pairs, respectively. The average spatial distance <*R*> of a locus pair A and B was calculated by using the time average <*R(t)*>_t_ of the spatial distance |***P****_A_(t)-**P**_B_(t)*| over 30 time frames, followed by cell population average, namely <*R*>=<<*R*>_t_>_cell_ (Tark-Dame et al., 2011). The temporal variation δ*R(t)* of the spatial distance of a locus pair was calculated by the absolute deviation of spatial distance at time *t* from the time average of the spatial distance, δ*R(t)*=|*R(t)*−<*R*>_t_|. The cell-to-cell variation of spatial distance of locus pairs at each cell cycle stage were analyzed and plotted according to the distribution of <*R*>_t_ over cell populations (Fig. S5). The temporary variation of locus pairs at each cell cycle stage was analyzed and plotted according to the distribution of δ*R(t)* over cell cycle stages (Fig. S6). The effective diffusion constant (*D_eff_*) of short-time locus dynamics was estimated using (Schuss, 2010):

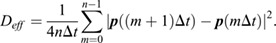


The short-time locus dynamics can be modeled using the Langevin equation (Vivante et al., 2020):

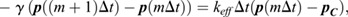
where *k_eff_* is the effective spring constant measuring the effective external force applied on the locus, and γ is the viscous coefficient given by the Einstein relation γ=*k*_B_T/*D_eff_*. The *k_eff_* value can be obtained from the slope of the linear fitting of the step size ***p***((*m*+1)Δ*t*)−***p***(*m*Δ*t*) of locus dynamics and its relative position ***p***(*m*Δ*t*)−***p***_***C***_ to the centroid of the locus trajectory. To calculate the tethering spring constant *k_t_* and associated position c_t_, we consider the Rouse polymer model with an effective spring constant K*_R_*. For two labeled loci located at c_1_ and c_2_, and one tethered locus located at c_t_ (Fig. 6E), the effective spring constant *k_eff,i_* of locus c_i_ is determined by (Amitai et al., 2015):

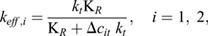
where Δ*c*_*it*_=|*c*_*i*_−*c*_*t*_| is the distance between the position of tethered c_t_ and labeled locus c_i_. By using the effective spring constants *k_eff,1_* and *k_eff,2_* measured by the locus-tracking data at positions c_1_ and c_2_, the chromosome effective spring constant K*_R_* can be determined by K*_R_*=(Δc_1*t*_−Δc_2*t*_) *k_eff,1_k_eff,2_*/(*k_eff,2_*−*k_eff,1_*). Assuming that c_1_<c_2_<c_t_, the tethering spring constant k_t_ and c_t_ can be solved by the linear regression of 1/*k*_*eff*,*i*_ and 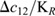.

All analyses were performed by *Mathematica* and graphs were generated by *OriginPro* (OriginLab version 2019b). All box-and-whisker plots were generated by using the default setting of the *OriginPro*. Each box has the average value marked by a line and spans from first to last quartiles, and whisker length was determined by the outermost data points that fall within the upper inner fence and lower inner fence (a coefficient=1.5). Significance tests were performed using one-way ANOVA function in OriginPro. ‘ns’ indicates statistically not significant data (*P*>0.05), statistically significant data are indicated as *P<0.05, **P<0.005 and ***P<0.0005. Pearson's correlation coefficient was calculated using OriginPro by using the default Pearson's Correlation function.

## Supplementary Material

10.1242/joces.260137_sup1 Supplementary information
