# Supplementary material for: Single-chromosome dynamics reveals locus-dependent dynamics and chromosome territory orientation
Source: J Cell Sci. 2023 Feb 27;136(4):jcs260137. doi: 10.1242/jcs.260137 (PMC10022681; doi:10.1242/jcs.260137)
Supplement: Supplementary information [file joces-136-260137-s1.pdf]

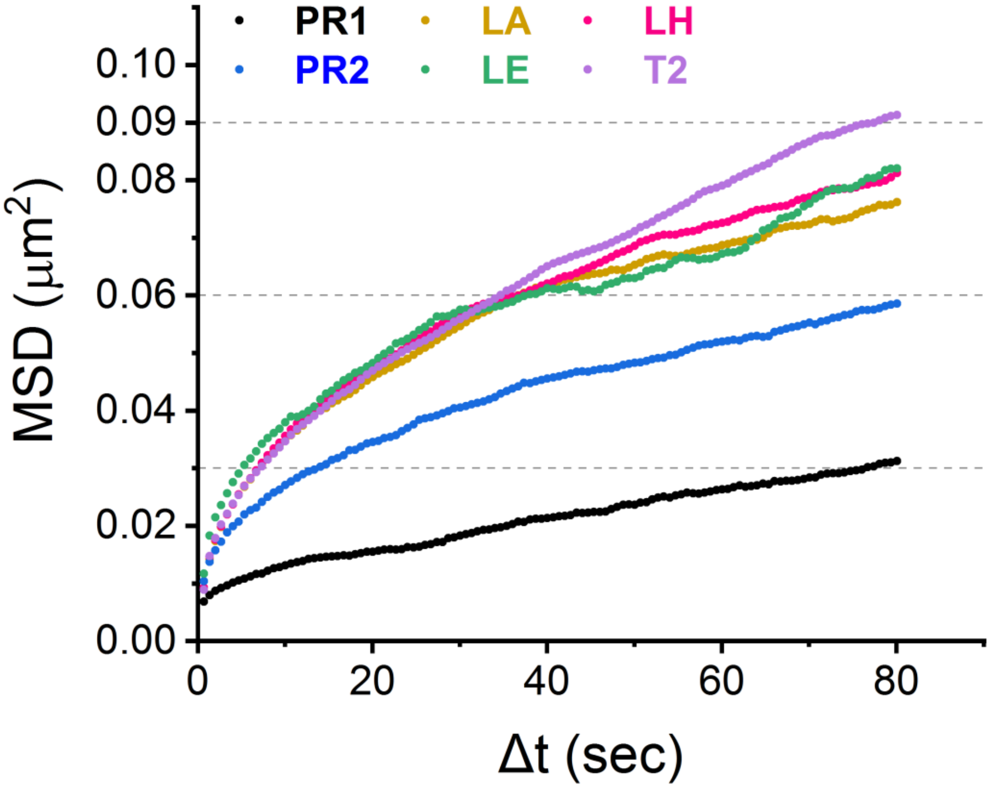

Fig. S1. Power-law plot of MSD curves. MSD curves of genomic loci (n = 28 trajectories for PR1, 52 trajectories for PR2, 27 trajectories for LE, 77 trajectories for LH, 77 trajectories for LA, and 127 trajectories for T2).

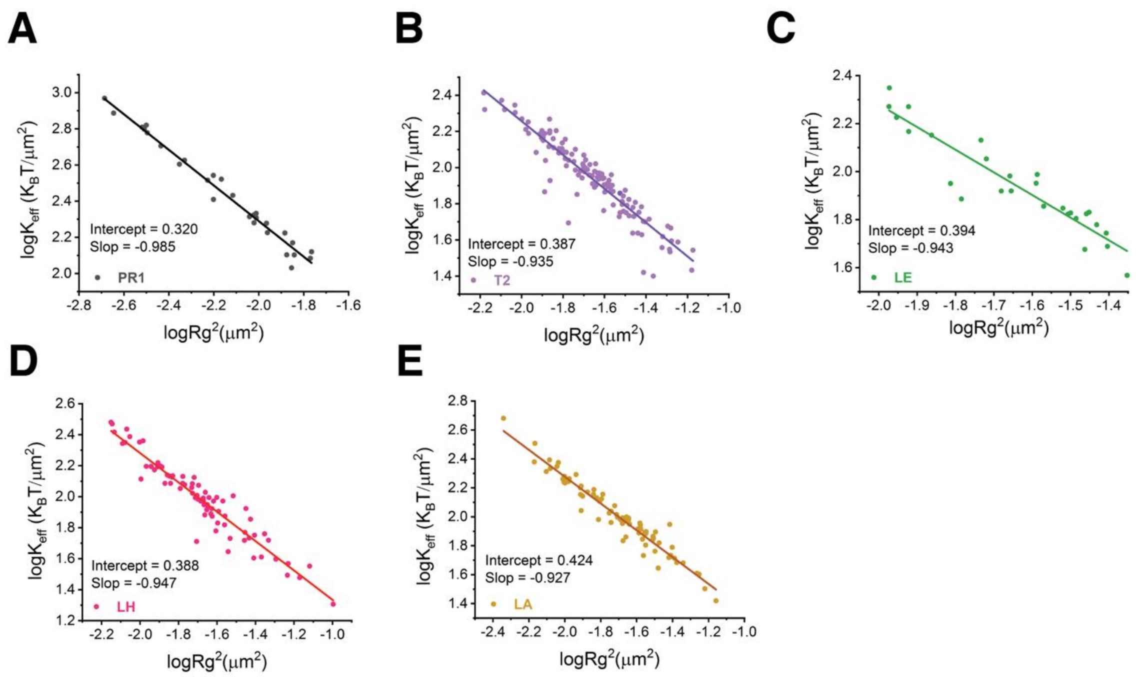

Fig. S2. Effective force constraints locus dynamics. (A)-(E) Log-log scatter plot of effective spring constants and loci territories for PR1, T2, LE, LH and LA. The data were fitted by linear regression (the solid lines) (n = 28 trajectories for PR1, 52 trajectories for PR2, 27 trajectories for LE, 77 trajectories for LH, 77 trajectories for LA, and 127 trajectories for T2).

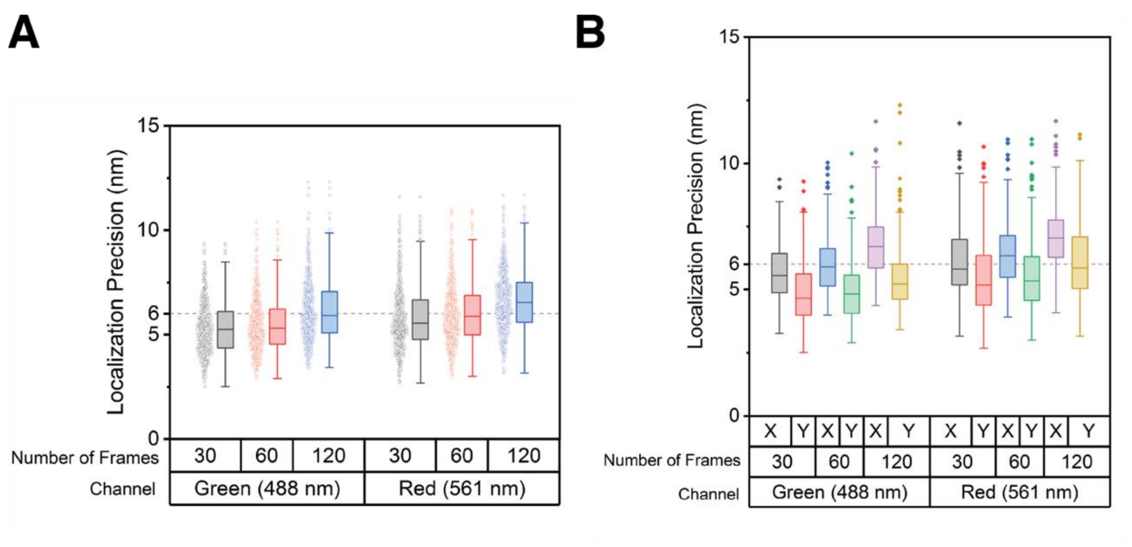

Fig. S3. Localization precision in seconds. (A) Localization precision of the green (488 nm excitation) and red (561 nm excitation) channels in 4 seconds (30 frames), 8 seconds (60 frames), and 16 seconds (120 frames) using 100 nm coverslip-immobilized beads (n = 220). (B) Localization of the x and y axes within 16 seconds.

Journal of Cell Science • Supplementary information

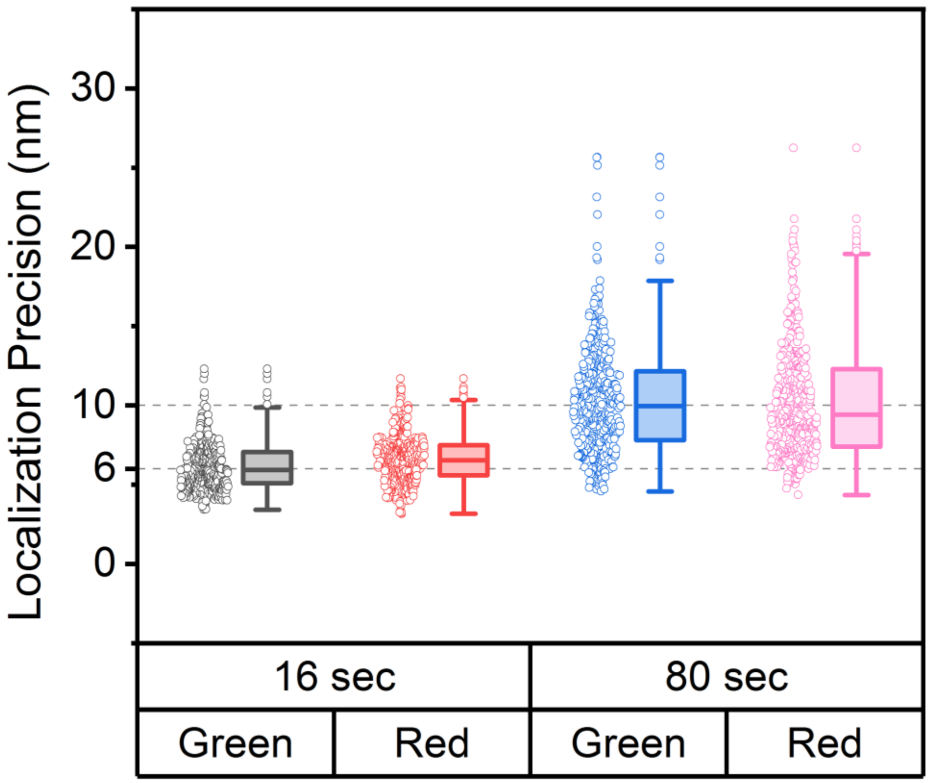

Fig. S4. Localization precision over a minute. Localization precision of the green (488 nm excitation) and red (561 nm excitation) channels in 16 seconds (120 frames, n = 220) and 80 seconds (120 frames, n = 218) using 100 nm coverslip-immobilized beads.

Journal of Cell Science • Supplementary information

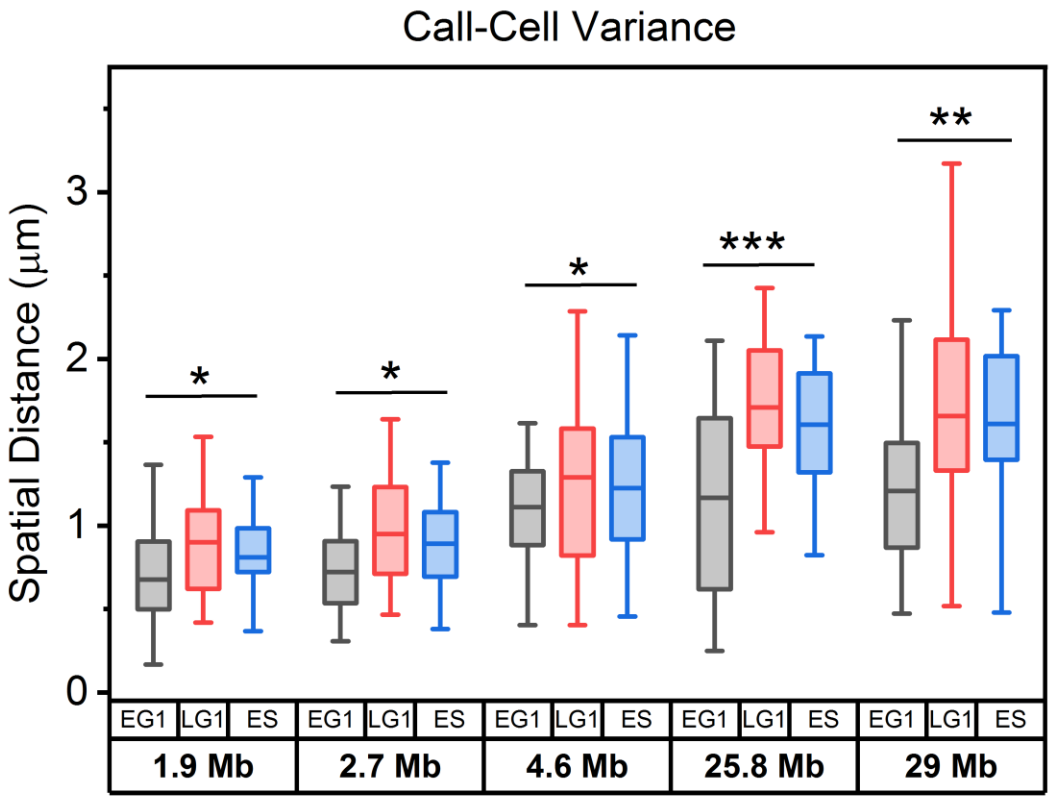

Fig. S5. Cell-to-cell spatial distance distribution of pairs of loci during the cell cycle. Boxplot of spatial distance distribution for each pair of loci at each cell cycle stage (from left to righ, N_{cell} = 30 (EG1), 27 (LG1), and 26 (ES) for 1.9 Mb locus pair LH/LA, N_{cell} = 34 (EG1), 25 (LG1), and 26 (ES) for 2.7 Mb locus pair LA/T2, N_{cell} = 25 (EG1), 29 (LG1), and 27 (ES) for 4.6 Mb locus pair LH/T2, N_{cell} = 27 (EG1), 26 (LG1), and 28 (ES) for 25.8 Mb locus pair LE/T2, N_{cell} = 25 (EG1), 29 (LG1), and 29 (ES) for 29 Mb locus pair LD/T2). Statistical significance for data from each pair of loci was tested by one-way ANOVA test. * $p < 0.05$, ** $p < 0.005$, *** $p < 0.0005$.

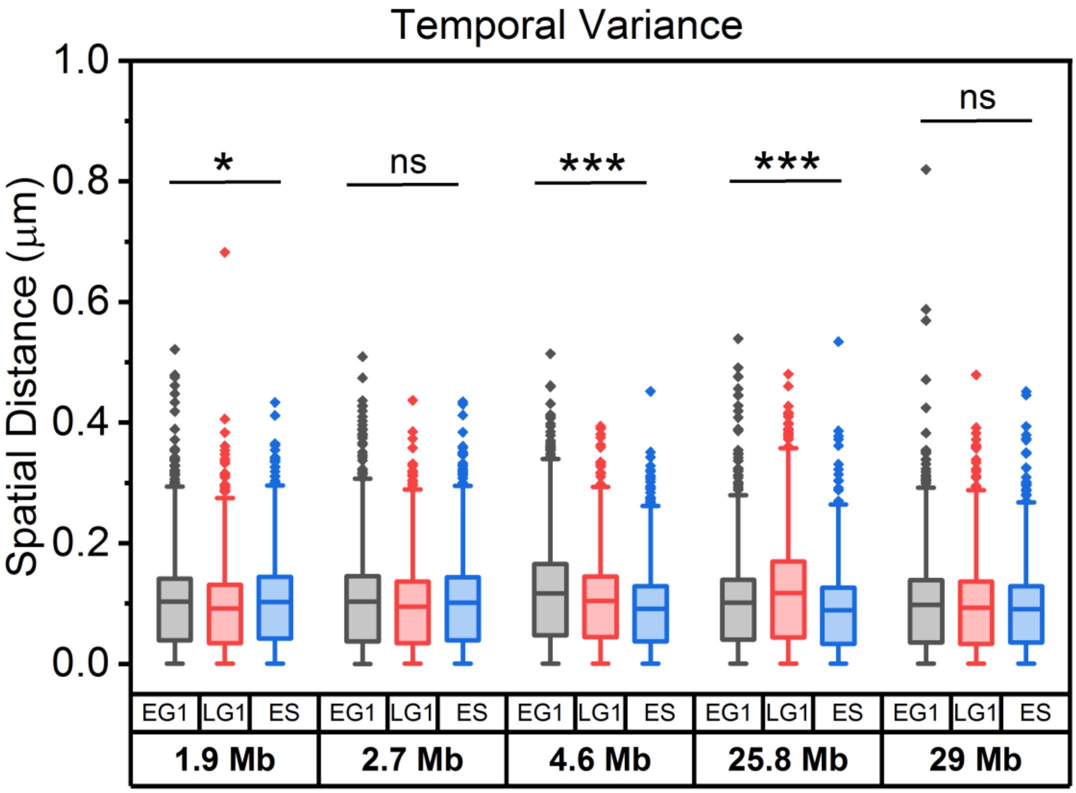

Fig. S6. Temporal variation of the spatial distance of pairs of loci during the cell cycle. Boxplots of temporal variation of spatial distance for each pair of loci at each cell cycle stage (from left to righ, n = 900 (EG1), 810 (LG1), and 780 (ES) for 1.9 Mb locus pair LH/LA, n = 1020 (EG1), 750 (LG1), and 780 (ES) for 2.7 Mb locus pair LA/T2, n = 750 (EG1), 870 (LG1), and 810 (ES) for 4.6 Mb locus pair LH/T2, n = 810 (EG1), 780 (LG1), and 840 (ES) for 25.8 Mb locus pair LE/T2, n = 750 (EG1), 870 (LG1), and 870 (ES) for 29 Mb locus pair LD/T2). Statistical significance for data from each pair of loci was tested by one-way ANOVA test. * $p < 0.05$, ** $p < 0.005$, *** $p < 0.0005$.

Journal of Cell Science • Supplementary information

Table S1. Compaction exponents extracted from locus pairs

Genomic Locus Pair	N (cells)	Compaction Exponent (δ)	Cell Cycle Phase
1.93 Mb LH/LA	36	N/A	Asynchronous
2.69 Mb LA/T2	41	N/A	Asynchronous
4.62 Mb LH/T2	41	0.40 ± 0.02	Asynchronous
25.82 Mb LE/T2	27	0.18 ± 0.04	Asynchronous
29.05 Mb PR2/T2	28	0.20 ± 0.03	Asynchronous
29.05 Mb PR2/T2	25	0.17 ± 0.06	EG1
29.05 Mb PR2/T2	29	0.21 ± 0.03	LG1
29.05 Mb PR2/T2	27	0.22 ± 0.03	ES

Table S2. Biophysical parameters extracted from single locus trajectories

Genomic Locus	MSD Exponent (β)	Radius (μm)	D_{app} (μm/sec$^{\beta}$)	N‡
T2	(4.57 ± 0.02) × 10^{-1}	(1.52 ± 0.37) × 10^{-1}	(3.02 ± 0.10) × 10^{-3}	127
LA	(4.04 ± 0.03) × 10^{-1}	(1.46 ± 0.42) × 10^{-1}	(3.36 ± 0.16) × 10^{-3}	77
LH	(4.17 ± 0.02) × 10^{-1}	(1.51 ± 0.46) × 10^{-1}	(3.35 ± 0.11) × 10^{-3}	77
LE	(3.55 ± 0.04) × 10^{-1}	(1.54 ± 0.34) × 10^{-1}	(4.18 ± 0.20) × 10^{-3}	27
PR2	(3.59 ± 0.01) × 10^{-1}	(1.28 ± 0.32) × 10^{-1}	(2.98 ± 0.05) × 10^{-3}	52
PR1	(3.57 ± 0.07) × 10^{-1}	(0.88 ± 0.26) × 10^{-1}	(1.47 ± 0.15) × 10^{-3}	28
IDR1*	(3.41 ± 0.08) × 10^{-1}	(1.33 ± 0.51) × 10^{-1}	(5.31 ± 0.12) × 10^{-3}	7
IDR2*	(3.61 ± 0.08) × 10^{-1}	(1.52 ± 0.33) × 10^{-1}	(4.19 ± 0.10) × 10^{-3}	19
IDR3*	(3.64 ± 0.03) × 10^{-1}	(1.48 ± 0.36) × 10^{-1}	(4.86 ± 0.05) × 10^{-3}	49
TCF3*	(4.60 ± 0.05) × 10^{-1}	(1.28 ± 0.44) × 10^{-1}	(2.88 ± 0.04) × 10^{-3}	12
IDR4*	(4.41 ± 0.07) × 10^{-1}	(1.58 ± 0.52) × 10^{-1}	(4.16 ± 0.08) × 10^{-3}	11

*These data were adopted from (Ma et al., 2019). ‡Number of trajectories

Table S3. Biophysical parameters of transcription inhibition experiments

Genomic Locus	DRB Treatment	MSD Exponent (β)	Radius (μm)	D_{app} (μm/sec$^{\beta}$)	N‡
CYP4F12	-	(3.47 ± 0.02) × 10^{-1}	(1.52 ± 0.37) × 10^{-1}	(3.09 ± 0.06) × 10^{-3}	40
CYP4F12	+	(3.31 ± 0.02) × 10^{-1}	(1.28 ± 0.32) × 10^{-1}	(3.59 ± 0.07) × 10^{-3}	31
ZNF358	-	(3.28 ± 0.02) × 10^{-1}	(0.88 ± 0.26) × 10^{-1}	(2.26 ± 0.07) × 10^{-3}	41
ZNF358	+	(3.42 ± 0.03) × 10^{-1}	(1.58 ± 0.52) × 10^{-1}	(4.02 ± 0.11) × 10^{-3}	56

‡Number of trajectories

Journal of Cell Science • Supplementary information

Table S4. Average effective spring and diffusion constants of single locus

Genomic Locus	K_{eff} ($K_BT/\mu m^2$)	D_{eff} ($\mu m^2/s$)
T2	97.35	0.00332
LA	118.13	0.00333
LH	111.86	0.00350
LE	97.73	0.00418
PR2	153.78	0.00386
PR1	336.26	0.00251

Table S5. Genome coordinate of human chromosome-19-specific repeats

	Label	Start	End
1	PR1	21049341	21099156
2	PR2	30002646	30005739
3	LE	33230168	33231522
4	LH	54422568	54428888
5	LA	56361126	56363117
6	T2	59050388	59054262
7	*CYP4F12*	16042336	16044890
8	*ZNF358*	7515015	7516031

Journal of Cell Science • Supplementary information

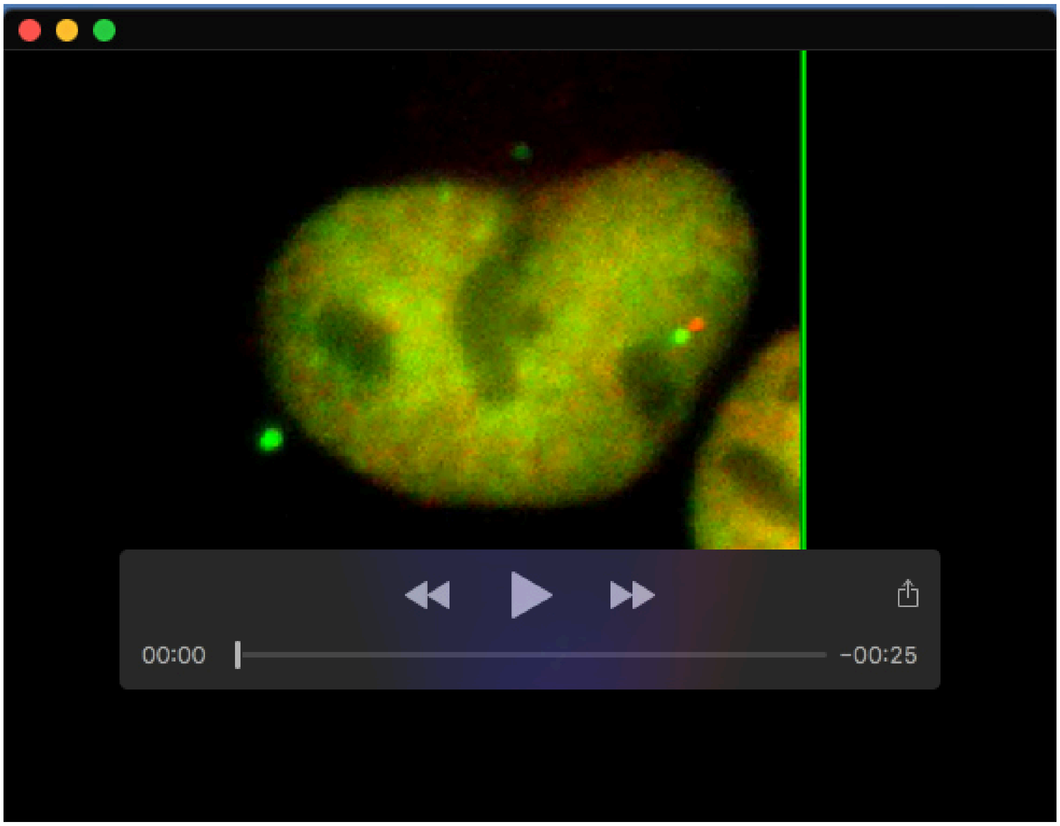

Movie 1. This movie shows a typical movement of the locus pair LH/T2 in the U2OS cell nucleus recorded over 80 seconds (120 image frames). LH is labeled in red by Halo tag-JF549 and T2 is labeled by GFP. The movie play rate is 30 Hz. The trajectories of loci in this movie are shown in Figure 3A.

References

Ma, H., Tu, L. C., Chung, Y. C., Naseri, A., Grunwald, D., Zhang, S. and Pederson, T. (2019). Cell cycle- and genomic distance-dependent dynamics of a discrete chromosomal region. *J Cell Biol* **218**, 1467-1477.

Journal of Cell Science • Supplementary information
