# Supplementary material for: Single-chromosome dynamics reveals locus-dependent dynamics and chromosome territory orientation
Source: J Cell Sci. 2023 Feb 27;136(4):jcs260137. doi: 10.1242/jcs.260137 (PMC10022681; doi:10.1242/jcs.260137)
Supplement: Supplementary file 1 [file joces-136-260137_review_history.pdf]

Single-chromosome dynamics reveals locus-dependent dynamics and chromosome territory orientation

Yu-Chieh Chung, Madhoolika Bisht, Jenna Thuma and Li-Chun Tu
DOI: 10.1242/jcs.260137

Editor: David Glover

Review timeline

Original submission:	16 April 2022
Editorial decision:	11 July 2022
First revision received:	16 November 2022
Editorial decision:	10 January 2023
Second revision received:	17 January 2023
Accepted:	19 January 2023

Original submission

First decision letter

MS ID#: JOCES/2022/260137

MS TITLE: Single Chromosome Dynamics Reveals Locus-Dependent Dynamics and Chromosome Territory Orientation

AUTHORS: Yu-Chieh Chung, Madhoolika Bisht, and Li-Chun Tu
ARTICLE TYPE: Research Article

We have now reached a decision on the above manuscript.

To see the reviewers' reports and a copy of this decision letter, please go to: https://submit-jcs.biologists.org and click on the 'Manuscripts with Decisions' queue in the Author Area. (Corresponding author only has access to reviews.)

As you will see, the reviewers raise a number of substantial criticisms that prevent me from accepting the paper at this stage. They suggest, however, that a revised version might prove acceptable, if you can address their concerns. If you think that you can deal satisfactorily with the criticisms on revision, I would be pleased to see a revised manuscript. We would then return it to the reviewers.

Please ensure that you clearly highlight all changes made in the revised manuscript. Please avoid using 'Tracked changes' in Word files as these are lost in PDF conversion.

I should be grateful if you would also provide a point-by-point response detailing how you have dealt with the points raised by the reviewers in the 'Response to Reviewers' box. Please attend to all of the reviewers' comments. If you do not agree with any of their criticisms or suggestions please explain clearly why this is so.

Reviewer 1

Advance summary and potential significance to field

© 2023. Published by The Company of Biologists under the terms of the Creative Commons Attribution License (https://creativecommons.org/licenses/by/4.0/).

In their manuscript entitled "Single Chromosome Dynamics Reveals Locus-Dependent Dynamics and Chromosome Territory Orientation" Chung et al. use their previously published CRISPR-Sirius chromatin labeling approach (Ma et al., 2018) to image new genomic loci on human chromosome 19 with conventional fluorescence microscopy. From the obtained time-lapse videos, various biophysical parameters of loci are quantified such as the diffusion and radius of gyration. Relating the relative position of the imaged loci to the genomic distance, and quantifying the radius of gyration, measures of compaction of the various loci are obtained. Interestingly, all the imaged q-arm loci showed less compaction compared to their previously characterized p-arm loci (Ma et al., 2019), which correlates with an overall higher transcription level. This indicates and is aligned with the notion that transcriptionally more active regions of DNA are less compact and thus more accessible. Moreover, the authors detect a higher probability of q-arm loci to be in the periphery of the nucleus whereas the reference p-arm locus seemed more randomly located. Using the quantified parameters and a rouse model, the authors also show that interactions of the centromeric regions are stronger than the q-arm regions close to telomeres.

Comments for the author

All experiments and quantifications of this manuscript are carried out well, and the comparison of the newly imaged q-arm loci to the previous p-arm locus are interesting. However, we have some concerns about the novelty and significance of biological findings required for publication in the Journal of Cell Science. The previously published CRISPR-Sirius based labeling and imaging technique has been applied to new q-arm loci in this manuscript and the correlation between less compaction and higher transcriptional activity is interesting. But we'd like to encourage the authors to think about a straight forward experiment to obtain some new insights beyond the biophysical characterization. For instance, does compaction or other quantified parameters show a dependence on the cell cycle similar to Ma et al. 2019? Or can the authors externally manipulate chromatin organization e.g. by adding the drugs such as HDAC inhibitors to open chromatin or 1,6 hexanediol to close chromatin and see how the biophysical parameters they measure with changes in chromatin states.

Besides this major point there are a few smaller points that need to be addressed:

- Line 89: how is the "fluorescence intensity" stable for hours or what do the authors want to highlight? The authors image loci for minutes (e.g. Fig. 3) and bleaching is primarily dependent on the used excitation power.

- Line 167: the lowest number of copies in the table says 38 but the authors write 20.

- Related to our remark about cell cycle above, how well does the author's criterium of only observing 1 locus per nucleus represent the cell cycle and could a cell cycle state in which the locus is not replicated yet have a different organization of chromatin e.g. with respect to compaction of diffusivity measurements? This should be addressed or at least discussed.

- Line 217: the authors quantify the absolute diffusion of the respective loci. Could a quantification of the relative diffusion between each pair of loci yield similar insights e.g. in compaction and how would this compare the results from the absolute measurements?

- Line 219: The authors fit the MSD without constant offset, which represents the localization uncertainty. It should either be shown that this offset has no significant impact on the diffusion coefficient, or all MSD fits should be done with offset.

- Line 224: please include units

- Line 239: wrong reference to Fig. S3

- How is the localization uncertainty in Fig. S3 calculated? Please describe or provide a reference.

- Line 251-254: it is not clear exactly what the authors mean. I guess they mean the variability of quantified parameters. Please clarify.

© 2023. Published by The Company of Biologists under the terms of the Creative Commons Attribution License (https://creativecommons.org/licenses/by/4.0/).

- Fig. 2 captions (and wherever applicable): please indicate the number of cells used in the statistics.

- Fig. 1 Why is PR1 not shown? It would be more complete.

- Fig. 3: please use ln or log consistently and indicate units on all graphs.

- Fig. 4: Please clarify units of diffusion coefficient since the fit comes from a time with an exponent unequal to 1.

Reviewer 2

Advance summary and potential significance to field

The senior author of this manuscript Li-Chun Tu has set up a new lab working on Chromatin Organization and Dynamics in Live Cells in the Wexner Medial Center of the Ohio State University. Previously, Tu worked as postdoc in the Thoru Pederson lab, where she contributed essentially to the development of CRISPR-Sirius (Ma H, Tu, L.C. et al., 2018). I have never met Tu, but followed this work closely, because it opens avenues beyond the limited scope of studies of fixed cells. This method is “based on octet arrays of aptamers conferring both enhanced guide RNA stability and brightness of targeted loci.” CRISPR-Sirius has helped to overcome problems of guide RNA instability, which lowered genome labeling efficiency of such loci in Pederson’s original approach (Ma H et al. (2015) Multicolor CRISPR labeling of chromosomal loci in human cells. PNAS 112(10), 3002-3007). Ma et al. (2015) used human U2OS cells expressing dCas9-fluorescent proteins and cognate sgRNAs. In the present study, Chung et al. use living U2OS cells to study movements of paired genomic loci mapped along human chromosome 19 with various genomic distances (1.93 Mb (LH/A4), 2.69 MB (LA/T2), 4.62 Mb (LH/T2), 25.82 MB (LE/T2), and 29.05 MB (PR2/T2) (Table S1). Figure 1B presents the localization of the targeted loci along a schematic normal human chromosome 19. PR1 locates on the p-arm as part of (or close to) the pericentromeric heterochromatin, five additional loci map along the q-arm including PR2 at the q-arm site of pericentromeric region and T2 close to the q-telomer, while LE, LH and LA map along the q-arm at sites between PR2 and T2. This study extends a previous study of the dynamic behavior of 7 loci along the p-arm of chromosome 19 (Ma et al., 2019). For imaging of targeted pairs in dual-color with good fluorescence signals, the authors identified loci comprising low copies of repetitive sequences (from 20 to 160 copies) (Table 1). Using RNA-seq they confirmed that these loci map either within intergenic regions or represent inactive genes with undetectable transcripts per million (TPM) or TPM smaller than 0.06 (Table 1). Live cell imaging was performed with conventional resolution. When reading real-time single-molecule microscopy in the Abstract, I initially confused this term with single molecule localization microscopy (SMLM), a method of super-resolved fluorescent microscopy based on the localization of blinking events from individual fluorophores. In fact, the authors visualized not single molecules, but clusters of many fluorescent proteins targeted to a given locus of repetitive sequences. The localization precision was measured by capturing 120 frames of 0.1 μm coverglass immobilized TetraSpeck fluorescent microspheres ($n = 220$ for 16 seconds and $n = 218$ for 80 seconds) at 100 ms exposure time and calculating the standard deviations, a method developed by Jeff Gelles (Gelles et al., 1988). The lateral localization precision determined by 100 nm coverslip-absorbed Tetraspeck beads was ~ 6 nm in 16 seconds (Figure S3) and ~10 nm in 80 seconds (Figure S4). In Spectral Precision distance microscopy (SPDM), this high localization precision also allows distance measurements between point like targets emitting light at different wavelength (Cremer et al. Superresolution imaging of biological nanostructures by spectral precision distance microscopy. Biotechnol. J. 2011 6, 1037–1051; DOI 10.1002/biot.201100031). Using the SPDM approach, Chung et al. were able to record live cell trajectories of pairs of false colored green and red loci, respectively, with a localization precision well below the Abbe limit during 80 seconds (Figure 3B). Paired loci were selected in individual light optical sections on the criterion that both loci were close to each other indicating their presence on the same chromosome 19 territory (Note, however, that intact chromosome 19 copies are lacking in U2OS cell, see below major comments, point 1). Based on these trajectories, Chung et al. calculated ensemble-averaged MSD curves, effective diffusion constants and trajectory radii for all studied loci (Figure 3). Figure 4 presents a correlation analysis between normalized radial distances between paired loci and the effective diffusion constants. This analysis appears of high

© 2023. Published by The Company of Biologists under the terms of the Creative Commons Attribution License (https://creativecommons.org/licenses/by/4.0/).

quality with the limitation that only a 2D analysis of individual light optical sections was performed which does not take into consideration movements in the 3rd dimension. Such movements were apparently small in the studied light optical sections, which showed both signals with comparable intensities during the 80 second observation period. The desirable investigation of much more extended periods was likely not possible due to bleaching.

Chung et al. report tighter packing of the chromosome 19 q arm compared with the previously studied p-arm in line with the analysis of U2OS RNA-seq data which show that the number of active genes on the p arm is ~10% more than the active genes on the q arm. The authors infer that more intense transcriptional activities on the more gene-dense p-arm could lead to a more extended and relaxed chromatin configuration based on potentially lower nucleosome density and more active transcription factor interactions.

Major comments:

1. The human osteosarcoma cell line U2OS is highly aneuploid. In karyotypes of U2OS cells studied with multicolor-painting of all chromosomes, every homolog is affected by clonal structural or numerical aberrations (see Figure 1 in Raftopoulou et al. (2020) Cancers 12, 591; doi:10.3390/cancers12030591). In our own unpublished U2OS multicolor paint karyotypes we could not detect any intact copy of chromosome 19. The karyotypic evolution during the propagation of the widely distributed U2OS cell lines has resulted in karyotypic diversity (see Figure 1 in Raftopoulou et al., 2020). We cannot exclude the possibility that the line used by Chung et al., may contain a normal copy of chromosome 19, but in the absence of a thorough chromosome analysis it is not possible to draw conclusions about the actual intranuclear arrangements of the p- and q-arm of an intact copy of chromosome 19 in U2OS cell nuclei, as claimed by the author's proposal of a "guided radial model".

2. To measure the compaction of the chromosome 19 q arm, the authors plotted average spatial distances of loci pairs against their genomic distance (Figure 2A). The compaction level of chromatin was determined by the scaling exponent (d, compaction exponent) of the power-law relationship between the spatial distance and genomic distance of loci pairs (Tark-Dame et al., 2011). The well-known compaction difference between extended DNA and the DNA packaged within a chromosome is on the order of several thousand-fold. Such values, however, do not inform us about three-dimensional, local compaction differences between chromatin harboring active and inactive genes, and dynamic 4D compaction changes, when inactive genes become active and vice versa. To answer questions of a possible relationship between local 3D chromatin compaction and the accessibility of DNA for transcription factors or whole transcription machineries, the compaction of the studied loci per se is irrelevant. Instead we need to know the 3D compaction and accessibility of the DNA landscape expanding between given pairs of targeted loci.

4. In Figure 3K, the authors present three models (Spaghetti, Ordered radial and Guided radial) of chromosome 19 long arm orientation and locus localization in the U2OS cell nucleus. The Spaghetti Model argues for a random organization with no tendency of nuclear radial distribution on any genomic loci. The Ordered-radial Model proposes a gradient preference of nuclear radial distributions according to their genomic locations along the chromosome and some level of rigidity to maintain the ordered structure. The authors argue that both the Spaghetti Model and the Ordered-radial Model "were not observed in our data". Instead, they claim that the data support the Guided-radial Model with a preferred radial position of loci and the orientation of the chromosome 19 q arm in live cells (Figure 6C). I lack expertise in polymer models of chromatin and cannot follow the author's chain of theoretical reasoning to distinguish between the three models and favor the third one. The reference "Doi, M. and Edwards, S. F. The theory of polymer dynamics" is incomplete (Oxford University Press 1986). The cartoon in Figure 6C depicts the assumed folding of the higher order chromatin fiber of the chromosome 19 p-arm by a line, but it is not indicated how thick this chromatin fiber may actually be? At face value, all three models suggest a wide space between the depicted chromatin fiber, but in the absence of evidence for the real organization of this fiber, it is not clear what the higher order chromatin organization assumed by the authors means for the accessibility of active and inactive genes at the DNA level? For lack of expertise, I cannot judge the author's theoretical reasoning of how local interactions constrain the locus dynamics based on the short-time locus dynamics of CRISPR440 Sirius locus-tracking data (lines 437-454). The authors describe distinct distributions of the effective diffusion constant Deff

© 2023. Published by The Company of Biologists under the terms of the Creative Commons Attribution License (https://creativecommons.org/licenses/by/4.0/).

for the studied loci (Figure 4D-I, right-hand-side histograms). PR1 has the narrowest Deff distribution among loci. On the q-arm, PR2 and T2 have narrower Deff distributions compared to other loci." Arguably, the range of Deff distribution may reflect the frequency of locus-local environment interactions, while the cross-linking of chromatin may contribute to the diffusion exponent of subdiffusive long-term locus dynamics (Amitai et al., 2017). According to the author's interpretation, the narrow Deff distributions on loci at pericentromeric and near telomeric regions suggest the high frequency of locus-local environment interactions, such as locus-nuclear landmark interactions. I consider this this interpretation reasonable.

Comments for the author

I recommend a major revision of the present manuscript, presenting the dynamic aspects of the investigated loci, but with a clear indication of the special cytogenetic features and restrictions of the U2OS cancer cell line. Locus-local environment interactions observed in U2OS cell nuclei do not represent interactions occurring along structurally normal chromosome 19 territories, but rather interactions of these loci in rearranged chromosomes carrying segments of the long arm of chromosome 19. Multiple translocations with other chromosomes in U2OS cell lines raise the question, whether locus-local environment interactions in rearranged chromosomes differ from interactions in structurally normal chromosomes 19. For a direct proof of the guided radial orientation of intact chromosome 19 territories (proposed in Figure 6C), it would be necessary to repeat the whole set of experiments with another human cell line, carrying at least one intact copy of chromosome 19, ideally employing human diploid cells. Such a study seems not feasible within the timescale of a normal revision, The construction of additional cell lines expressing the necessary dCas9-fluorescent proteins and cognate sgRNAs is a challenge with the risk that protocols used for the establishment of the respective U2OS cells (Ma et al., 2015) may fail or need major modifications.

In the Abstract, Chung et al. state that "dynamic chromatin organization instantly influences DNA accessibility through modulating local macromolecular density and interactions, driving changes in transcription activities. … However, the dynamics, nuclear orientation, and compaction of sub-regions along a single chromosome are not well-understood. " These sentences whet the appetite of readers eager to learn more about essential relationships between local differences of chromosome compaction and DNA accessibility, but the manuscript in its present form does not fulfill this expectation.

I add some suggestions for future studies, which should be considered by the authors as my personal hints for a strategy to further resolve the major biological problem of chromatin compaction and accessibility, not as a to-do list, which needs to be fulfilled in this reviewer's opinion for the present publication.
For a meaningful study of dynamic changes of the 3D chromatin compaction of active and inactive genes, it seems necessary to design a CRISPR-Sirius strategy for live cell studies of loci carrying either active genes or inactive genes. An ideal model case should provide a gene, whose transcriptional activity can be experimentally modulated from a silent to a transcriptionally highly active state or vice versa.

It seems highly advisable for future studies to combine the present live cell approach sequentially with super-resolved microscopy of the fixed cell specimens. Such sequential analyses provide the possibility to study live-cell trajectories of loci with the surrounding, super-resolved chromatin landscape.

Direct studies of the compaction of active and inactive genes with the CRISPR-Sirius approach may further require a major improvement of the sensitivity of this method. Such an improvement would also be of great benefit to extend the observation period of signals (currently 80 sec).

<u>Reviewer 3</u>

Advance summary and potential significance to field

The use of the CRISPR cas9 methodology has made chromatin and chromosome dynamic as an experimental system much more accessible. I appreciate there is a large amount of work to do this.

© 2023. Published by The Company of Biologists under the terms of the Creative Commons Attribution License (https://creativecommons.org/licenses/by/4.0/).

My feeling with this piece of work is that it is interesting up to a point with respect to behaviour of chromatin in localised area on chromosome 19, a very active chromosomes but doesn't quite go far enough with say comparisons within the genome, within the cell cycle, other cell types, changes in transcription etc. The authors should look and cite papers where fish probes have been radially localised and compare their work to this - centromeric and telomeric regions within territories have been positioned in the past. Furthermore, the earlier work of the Bickmore lab for chromosome 19 should be included. should be cited since they did so much work initially on chromosome 19.

Comments for the author

I understand that there are some comparisons along the arms and between the arms of chromosome 19 and it is interesting to see the difference in chromatin dynamics around the centromere for chr 19 but the study doesn't feel like a significant enough study when compared to Ma et al., 2019 to presently publish in JCS. One can only make deductions really for chromosome 19 and not generally about other chromosomes in the genome without further study.

Data were - data the word is plural

21 trajectories is quite a low number for one of the data sets.

Comparing chromosome shapes to orange or lemon is not helpful and I wasn't quite sure what this meant. There are more scientific terms that can be used.

First revision

Author response to reviewers' comments

A point-by-point response to the reviewer's comments

Reviewer #1:
1) All experiments and quantifications of this manuscript are carried out well, and the comparison of the newly imaged q-arm loci to the previous p-arm locus are interesting. However, we have some concerns about the novelty and significance of biological findings required for publication in the Journal of Cell Science. The previously published CRISPR-Sirius based labeling and imaging technique has been applied to new q-arm loci in this manuscript and the correlation between less compaction and higher transcriptional activity is interesting. But we'd like to encourage the authors to think about a straight forward experiment to obtain some new insights beyond the biophysical characterization. For instance, does compaction or other quantified parameters show a dependence on the cell cycle similar to Ma et al. 2019? Or can the authors externally manipulate chromatin organization e.g. by adding the drugs such as HDAC inhibitors to open chromatin or 1,6 hexanediol to close chromatin and see how the biophysical parameters they measure with changes in chromatin states.

Response: We appreciate the encouraging comments about adding new insights beyond the biophysical characterization. To elevate the significance of the work, we have added two experiments: **(i) the chromosome 19 q arm compaction in different cell cycle stages; and (ii) the effects of transcription activities on genomic locus mobility**. We had to include an additional author because these experiments are time-consuming in that synchronizing cell cycle takes 2-3 days per sample, and the data from any one experiment must be analyzed in a process that takes several hours plus we have to perform many replicates of experiments to achieve the necessary statistical power. In the revised manuscript, we have shown that the compaction of chromosome 19 q arm is cell cycle-dependent but was very similar between late G1 (LG1) and early S (ES) phases (**Figure 1A**). A tighter compaction of chromosome 19 q arm at early G1 (EG1, $\delta = 0.17$) phase was detected when compared to the compaction levels in late G1 ($\delta = 0.21$) and early S stages ($\delta = 0.22$). As demonstrated by Abramo et al. [**2019** *Nat Cell Biol*], compartments started to develop 3-4 hrs after prometaphase by chromosome conformation capture techniques. Our results suggest, from the microscope perspective, that chromosome decondensation does not complete in early G1 phase, and remains tightly organized.

© 2023. Published by The Company of Biologists under the terms of the Creative Commons Attribution License (https://creativecommons.org/licenses/by/4.0/).

To investigate the effects of transcription activities on chromatin dynamics, we used the transcription inhibitor DRB (5,6-Dichloro-1-b-d-ribofuranosyl-benzimidazole) to block Pol II elongation. Two loci on chromosome 19 p arm were chosen based on the gene activities, a locus within gene *ZNF358* (zinc finger protein 358 with a TPM= 37.2 in U2OS) and another locus within the inactive gene *CYP4F12* as a control (TPM = 0 in U2OS). Without DRB treatment, CYP4F12 had a higher mobility when compared to the mobility of the locus within a transcribing gene *ZNF358*. The mobility of the gene-coded locus ZNF358 increased upon transcription inhibition by DRB while the mobility of the control locus CYP4F12 remained at a similar level (**Figure 1B (MSD) and 1C (gyration radius)**). Although increased nucleosome mobility was reported in cells with DRB treatment, tracking nucleosomes may not fully reflect transcription effects on the chromatin dynamics because nucleosomes are evited by the chromatin remodeler during transcription and are rarely found at a transcribing gene. Our data provide direct evidence to demonstrate the transcription inhibition of a gene leads to increased mobility on the active gene site but not the non-transcribing chromosomal regions on endogenous DNA sequences. We hope the revised manuscript with added biological insights would be suitable for the journal.

Figure 1

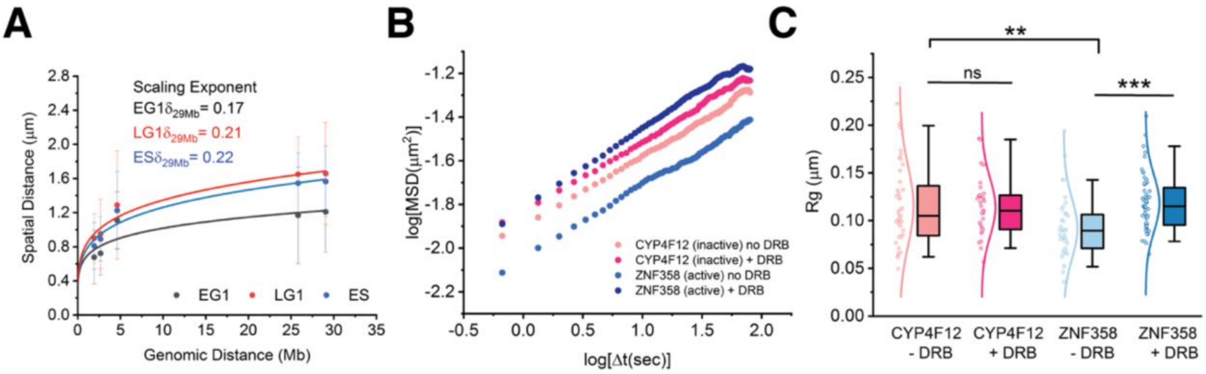

2) Line 89: how is the "fluorescence intensity" stable for hours or what do the authors want to highlight? The authors image loci for minutes (e.g. Fig. 3) and bleaching is primarily dependent on the used excitation power.

Response: We thank the reviewer's comments and apologize for the unclear sentence. We have tested the DNA binding kinetics of the CRISPR-gRNA complexes using FRAP (fluorescence recovery after photobleaching) in our previous work and found that the CRISPR-gRNA-DNA complexes are very stable [Ma et al. **2016** *JCB*]. The targeted CRISPR-gRNA complexes can remain bound for hours, which could contribute to the stability of fluorescence signals (e.g., fewer issues with blinking or unexpected fluorescence intensity changes). We removed the sentence in the revised manuscript to avoid misunderstanding.

3) Line 167: the lowest number of copies in the table says 38 but the authors write 20.

Response: The reviewer is correct about the lowest copy number. The locus with 20 copies was used in our previous work but not in this study. We have made the correction. The lowest number of copies in the revised manuscript is 29 for the genomic locus CYP4F12.

4) Related to our remark about cell cycle above, how well does the author's criterium of only observing 1 locus per nucleus represent the cell cycle and could a cell cycle state in which the locus is not replicated yet have a different organization of chromatin e.g. with respect to compaction of diffusivity measurements? This should be addressed or at least discussed.

Response: As shown in our new data, the cell cycle affects chromosome compaction in G1 phase but the compactions between the late G1 and early S were very similar. The effects on chromatin organization from DNA replication (during the S phase) are mostly from the cohesion process in which two sister chromatids are glued by acetylated cohesin complexes. Sister chromatid cohesion takes hours to complete and only breaks during anaphase (late mitosis) in the presence of active separase. To better show what we see in our system, a figure from our previous publication [Ma et

© 2023. Published by The Company of Biologists under the terms of the Creative Commons Attribution License (https://creativecommons.org/licenses/by/4.0/).

al. **2018** *Nature Methods*] is attached below (**Figure 2**). Before sister chromatid cohesion is stably in place during prophase, our CRISPR imaging captures replicated DNA as soon as they are generated in the mid-late S phase. However, tracking replicated loci is nontrivial because they constantly undergo "separate and merge" exercises during the cohesion process which we believe is not yet complete. We have excluded cells with multiple loci, and as a result, our data should highly represent the dynamics and chromatin organization of late G1 and early S phases.

Figure 2

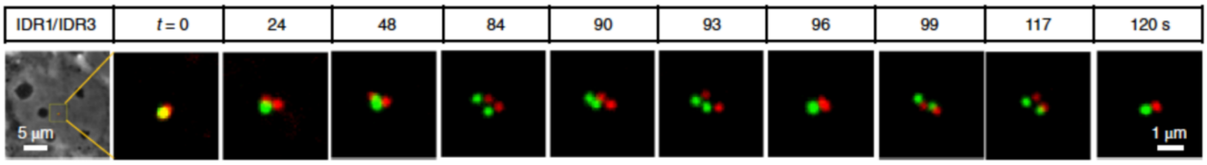

5) Line 217: the authors quantify the absolute diffusion of the respective loci. Could a quantification of the relative diffusion between each pair of loci yield similar insights e.g. in compaction and how would this compare the results from the absolute measurements?

Response: In our previous study[Ma et al. **2019** *JCB*], the relative diffusion between pairs of loci was used to detect dynamic chromatin organization at different genomic scales. Correlations between the relative diffusion and the chromatin compaction exist but it is not straightforward relationship. Additional input from the theoretical study and simulations of polymer models is necessary to clearly describe this relationship, which is beyond the scope of the paper. The relationship between the relative diffusion of pairs of loci and the absolute diffusion of individual loci is also not straightforward and is involved in the coupling between the movement of loci. We hope to elucidate the relationship among these quantities in the near future.

6) Line 219: The authors fit the MSD without constant offset, which represents the localization uncertainty. It should either be shown that this offset has no significant impact on the diffusion coefficient, or all MSD fits should be done with offset.

Response: We agree the offset in the MSD fitting is important to justify the localization uncertainty. We have followed the offset analysis used by Renner et al. [**2017** *Biophysical Journal* 113:2452]. In brief, the spot localization accuracy σ_{xy}^2 can be estimated by fitting the first 2-5 time points of the MSD plot with the equation $MSD(t) = 4D_{2-5}t + 4\sigma_{xy}^2$. Our localization uncertainty is 50 ± 5.8 nm (mean ± SD), which falls within a reasonable range for live-cell single-particle tracking. We have added this information in the Materials and Methods under Fluorescence Microscopy section.

	PR1	PR2	LE	LH	LA	T2	Average
$4\sigma_{xy}^2$ (μm^2)	0.0069	0.0092	0.0100	0.0105	0.0141	0.0101	
σ_{xy}	41.5 nm	48.0 nm	50.0 nm	51.2 nm	59.4 nm	50.2 nm	50 nm

7) Line 224: please include units

Response: We thank the reviewer's comments. These numbers are constant exponents of the power-law functions. They are dimensionless quantities.

8) Line 239: wrong reference to Fig. S3

Response: We appreciate the reviewer pointed out the wrong reference. We have revised Fig. S3 to Table S4.

9) How is the localization uncertainty in Fig. S3 calculated? Please describe or provide a reference.

Response: In the Materials and Methods under the "Fluorescence Microscopy" section, the measurement of localization uncertainty is described and a reference – Gelles et al., 1988 – had

© 2023. Published by The Company of Biologists under the terms of the Creative Commons Attribution License (https://creativecommons.org/licenses/by/4.0/).

been included. To be better explanatory about determining the localization uncertainty, we have added a sentence “The standard deviations from the repetitive measurement of beads were used to represent the localization uncertainty of our optical system.”

10) Line 251-254: it is not clear exactly what the authors mean. I guess they mean the variability of quantified parameters. Please clarify.

Response: We thank the reviewer’s comments. We have re-phrased the sentence as “To exclude the effects from transcription activities, we have used the genomic loci that are either located within intergenic regions or within genes that are not transcribed in U2OS cells (Table 1). The different mobility among genomic loci within a short-time period (Δt = 0.667 second) was mainly caused by the variability of chromatin-chromatin interactions (i.e., inter-locus interaction) and chromatin-environment (e.g, nuclear organelle) attachments.”

11) Fig. 2 captions (and wherever applicable): please indicate the number of cells used in the statistics.

Response: We thank the reviewer’s suggestion. The number of cells had been listed in Table S1 and we added the numbers in Fig. 2 captions in our revised manuscript.

12) Fig. 1 Why is PR1 not shown? It would be more complete.

Response: We thank the reviewer’s suggestion and have included an image in Fig. 1 that shows PR1 locus in the cell nucleus.

13) Fig. 3: please use ln or log consistently and indicate units on all graphs.

Response: We thank the reviewer’s suggestion and have revised the scales on all graphs.

14) Fig. 4: Please clarify units of diffusion coefficient since the fit comes from a time with an exponent unequal to 1.

Response: In Fig.4, the effective diffusion constants were calculated by using the MSD of short-time locus dynamics, and fitted by normal diffusion equation (linear function of time, instead of the power-law function of time). Therefore, the unit of the effective diffusion constant is μm^2/sec. This analysis has been used in published work by other groups (Amitai, A. et al. (2017) *Cell Rep* 18, 1200-1214; Vivante, A. et al. (2020). *Biophys J* 118, 2258-2267).

Reviewer #2:
We appreciate the reviewer’s in-depth comments and understanding of our work and encouragingly pointed out that our work opens avenues beyond the limited scope of studies from fixed samples. The reviewer raised a number of points that we appreciated and to which we respond as follows. We hope the revised manuscript with additional experiments and data would be suitable for the journal.

1) The human osteosarcoma cell line U2OS is highly aneuploid. In karyotypes of U2OS cells studied with multicolor-painting of all chromosomes, every homolog is affected by clonal structural or numerical aberrations (see Figure 1 in Raftopoulou et al. (2020) Cancers 12, 591; doi:10.3390/cancers12030591). In our own unpublished U2OS multicolor paint karyotypes we could not detect any intact copy of chromosome 19. The karyotypic evolution during the propagation of the widely distributed U2OS cell lines has resulted in karyotypic diversity (see Figure 1 in Raftopoulou et al., 2020). We cannot exclude the possibility that the line used by Chung et al., may contain a normal copy of chromosome 19, but in the absence of a thorough chromosome analysis it is not possible to draw conclusions about the actual intranuclear arrangements of the p- and q-arm of an intact copy of chromosome 19 in U2OS cell nuclei, as claimed by the author’s proposal of a “guided radial model”.
Response: We agree with the reviewer’s point that polyploidy is a typical phenotype of cancer cells. However, our U2OS cell line is from Dr. Thoru Pederson’s lab whose U2OS, purchased decades ago, is one of the very early tubes. In our previous work, we have bioinformatically determined that the U2OS p arm is diploid and the q arm could be either diploid or trisomic in U2OS. To further

© 2023. Published by The Company of Biologists under the terms of the Creative Commons Attribution License (https://creativecommons.org/licenses/by/4.0/).

demonstrate the chromosome 19 q arm copy number of our U2OS line, we counted chromosome 19 by using a painting guide RNA that targets ~836 copies on the chromosome 19 q arm and covers a genomic region of ~17 MB (Feng et al. 2020 Genome Research 21:296). In this experiment, copy number and integrity of chromosome 19 were directly visualized and counted in single cells. If multiple copies or abnormal amplification of chromosome 19 are present, we should easily see multiple well-separated signals (foci) in cells, but not the clustered signals representing intact chromosome 19 territories. Our data shows that ~63.8 % of cells are diploidy, 14.5 % of cells are triploidy, ~18.6 % of cells are haploidy, and ~2.8 % of cells were tetraploidy or more. For cells that showed one chromosome 19 q territory might simply have two homologs too close to be distinguishable. We do not exclude the possibility that part or all of these one chromosome 19 cells have two homologs. Therefore, our data demonstrated that the majority (> 77 %) of the total population has two or three intact copies of chromosome 19 q arm. We do not see significant karyotypic evolution issues on chromosome 19 in our U2OS line.

[NOTE: We have removed data that had been provided for the referees in confidence.]

2) To measure the compaction of the chromosome 19 q arm, the authors plotted average spatial distances of loci pairs against their genomic distance (Figure 2A). The compaction level of chromatin was determined by the scaling exponent (d, compaction exponent) of the power-law relationship between the spatial distance and genomic distance of loci pairs (Tark-Dame et al., 2011). The well known compaction difference between extended DNA and the DNA packaged within a chromosome is on the order of several thousand-fold. Such values, however, do not inform us about three-dimensional, local compaction differences between chromatin harboring active and inactive genes, and dynamic 4D compaction changes, when inactive genes become active and vice versa. To answer questions of a possible relationship between local 3D chromatin compaction and the accessibility of DNA for transcription factors or whole transcription machineries, the compaction of the studied loci per se is irrelevant. Instead, we need to know the 3D compaction and accessibility of the DNA landscape expanding between given pairs of targeted loci.

Response: Compaction exponents have been used to quantitatively compare the compaction levels among chromosomal domains in different epigenetic states, such as polycomb-repressed domains (Boettiger et al. **2016** *Nature* 529:418), x chromosome inactivation (Wang et al. **2016** *Science* 353:598), and lamina-induced chromosomal stretching (Sawh et al. **2020** *Mol Cell* 78:96). Our result is consistent with compaction exponent differences reported in previous studies in which actively transcribing chromosomal domains have larger compaction exponents compared to the compaction exponents of repressed chromosomal domains. To demonstrate the different dynamics between active and inactive genes mentioned by the reviewer, we performed the measurement of the dynamics on an actively-transcribing gene *ZNF358* (TPM = 37.2) and an inactive gene *CYP4F12* (TPM = 0). We further determined the locus dynamics under the transcription inhibitor DRB (5,6-Dichloro-1-b-d-ribofuranosyl-benzimidazole) treatment to block Pol II elongation. Without DRB perturbation, CYP4F12 had a higher mobility when compared to the mobility of the locus within a transcribing gene *ZNF358*. The mobility of the gene-coded locus ZNF358 increased upon transcription inhibition by DRB while the mobility of the control locus CYP4F12 remained at a similar level (**Figure 1B and 1C**). Although increased nucleosome mobility was reported in cells with DRB treatment, tracking nucleosomes may not reflect transcription effects on locus dynamics because nucleosomes are evited by the chromatin remodeler during transcription and are rarely found at a transcribing gene. Our data provide direct evidence to demonstrate the transcription inhibition of a gene leads to increased mobility on the active gene site but not the non-transcribing chromosomal regions on endogenous DNA sequences. We hope the revised manuscript with added biological insights would be suitable to the journal.

© 2023. Published by The Company of Biologists under the terms of the Creative Commons Attribution License (https://creativecommons.org/licenses/by/4.0/).

Figure 1

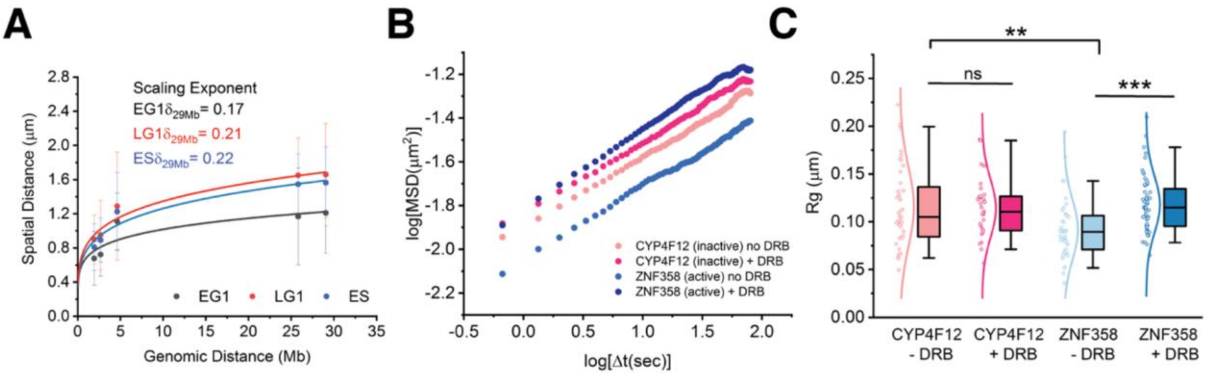

3) In Figure 3K, the authors present three models (Spaghetti, Ordered radial, and Guided radial) of chromosome 19 long arm orientation and locus localization in the U2OS cell nucleus. The Spaghetti Model argues for a random organization with no tendency of nuclear radial distribution on any genomic loci. The Ordered-radial Model proposes a gradient preference of nuclear radial distributions according to their genomic locations along the chromosome and some level of rigidity to maintain the ordered structure. The authors argue that both the Spaghetti Model and the Ordered-radial Model “were not observed in our data”. Instead, they claim that the data support the Guided-radial Model with a preferred radial position of loci and the orientation of the chromosome 19 q arm in live cells (Figure 6C). I lack expertise in polymer models of chromatin and cannot follow the author’s chain of theoretical reasoning to distinguish between the three models and favor the third one. The reference “Doi, M. and Edwards, S. F. The theory of polymer dynamics” is incomplete (Oxford University Press, 1986). The cartoon in Figure 6C depicts the assumed folding of the higher order chromatin fiber of the chromosome 19 p-arm by a line, but it is not indicated, how thick this chromatin fiber may actually be? At face value, all three models suggest a wide space between the depicted chromatin fiber, but in the absence of evidence for the real organization of this fiber, it is not clear what the higher order chromatin organization assumed by the authors means for the accessibility of active and inactive genes at the DNA level? For lack of expertise, I cannot judge the author’s theoretical reasoning of how local interactions constrain the locus dynamics based on the short-time locus dynamics of CRISPR440 Sirius locus-tracking data (lines 437-454). The authors describe distinct distributions of the effective diffusion constant Deff for the studied loci (Figure 4D-I, right-hand-side histograms). PR1 has the narrowest Deff distribution among loci. On the q-arm, PR2 and T2 have narrower Deff distributions compared to other loci.” Arguably, the range of Deff distribution may reflect the frequency of locus-local environment interactions, while the cross-linking of chromatin may contribute to the diffusion exponent of subdiffusive long-term locus dynamics (Amitai et al., 2017). According to the author’s interpretation, the narrow Deff distributions on loci at pericentromeric and near telomeric regions suggest the high frequency of locus-local environment interactions, such as locus-nuclear landmark interactions. I consider this interpretation reasonable.

Response: We appreciate the reviewer’s agreement with our data interpretation. Due to technical limitations, current live-cell imaging techniques cannot be used to precisely determine the thickness of interphase chromatin fibers which typically have sizes ranging from 11 nm to 33 nm. The chromosome organization models we proposed for the chromosome 19 q arm do not inform the exact thickness of a chromatin fiber but suggest the possible nuclear organization of the chromosome polymer. In combination with our localization and dynamic data, we excluded models that were not observed in our experiments. For example, randomly organized chromosomal domains are expected to have indistinguishable nuclear loci distribution and mobility. Random chromosome organization is a result of little or no attachment to nuclear organelles and weak inter- chromosomal interactions which results in weakly confine and restrict loci movement. On the other hand, to maintain a highly ordered chromosome organization, multiple strong interactions are required, which confine and reduce loci mobility. We found some loci, such as the locus at the pericentromeric and telomeric regions, were more confined and restricted than others, such as loci located in the interior arms, are not. This implies that chromosome organization is “guided” by certain interactions in the nucleus but still allows rearrangement to occur within the arms.

© 2023. Published by The Company of Biologists under the terms of the Creative Commons Attribution License (https://creativecommons.org/licenses/by/4.0/).

Reviewer #3:

I understand that there are some comparisons along the arms and between the arms of chromosome 19 and it is interesting to see the difference in chromatin dynamics around the centromere for chr 19 but the study doesn't feel like a significant enough study when compared to Ma et al., 2019 to presently publish in JCS. One can only make deductions really for chromosome 19 and not generally about other chromosomes in the genome without further study.

Response: The reviewer's point is appreciated. Our ongoing research is to expand the tools we developed on the investigation of human chromosomes other than chromosome 19 which we have started. Unlike fixed samples can be treated with hazardous chemicals or high-temperature DNA denaturation, live-cell samples can't be chemically treated and must be handled with care. We had explained in our previous work (but not yet in our submitted manuscript) that defining genomic repeats specific to a given chromosome is not straightforward. All targeted sequences have to fit the following criteria: (1) restricted to a particular chromosome; (2) located at certain locations (e.g., do not overlap and not too close to the confirmed sites); (3) have ideal copy numbers for the detection by our microscope; and (4) cell toxicity is non-detectable (e.g., not inducing or promoting cell death). This requires comprehensive bioinformatics mining of all the human repeats and control experiments of the actual locus brightness in a specific cell line. Therefore, repeating the entire dynamics study on other chromosomes will take at least another 6-9 months. To increase the significance of the work, we have added experiments that show how transcription affects genomic locus movements. This is one of the questions in which scientists in the field are highly interested; we have been asked many times in conferences and meetings about this. We reasoned that this will be good information to be included and elevates the significance of the work.
In this experiment, two genomic loci on chromosome 19 were chosen, a locus located within an active gene *ZNF358* with a TPM (transcripts per million) of 37.19 and a locus located within an inactive gene *CYP4F12* with a TPM of 0 in U2OS. Without DRB perturbation, CYP4F12 had a higher mobility when compared to the mobility of the locus within a transcribing gene *ZNF358*.
The mobility of the gene-coded locus ZNF358 increased upon transcription inhibition by DRB treatment while the mobility of the control locus CYP4F12 remained at a similar level (**Figure 1B and 1C**). Although increased nucleosome mobility was reported in cells with DRB treatment, tracking nucleosomes may not reflect transcription effects on locus dynamics because nucleosomes are evited by the chromatin remodeler during transcription and rarely found at a transcribing gene. Our data provide direct evidence to demonstrate the transcription inhibition of a gene leads to increased mobility on the active gene site but not the non-transcribing chromosomal regions on endogenous DNA sequences. In addition, we have measured the chromosome 19 q arm compactions in different cell cycle phases and demonstrated a tighter compaction in early G1 phase when compared to the compactions in late G1 and early S phases (**Figure 1A**). We hope the revised manuscript with added biological insights would be suitable to the journal.

Figure 1

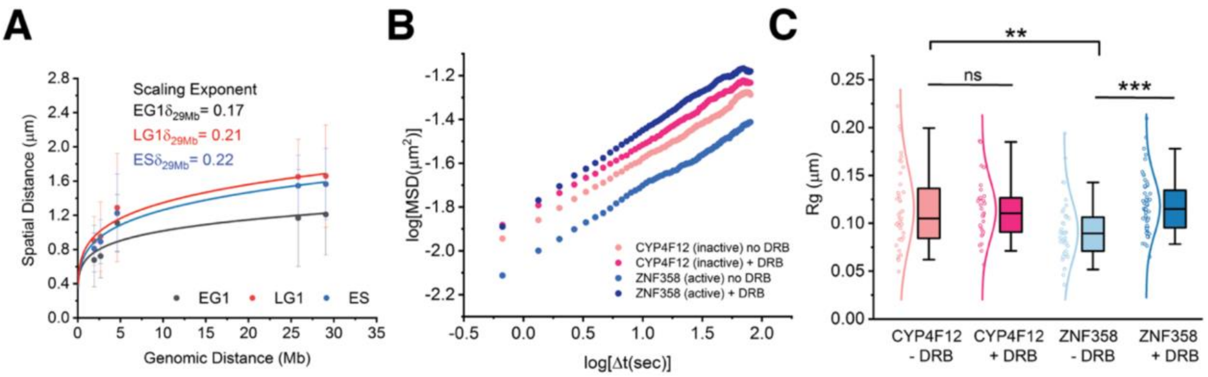

2) Data were - data the word is plural.

Response: We thank the reviewer's comment on the grammatical error and have corrected it in the revised manuscript.

© 2023. Published by The Company of Biologists under the terms of the Creative Commons Attribution License (https://creativecommons.org/licenses/by/4.0/).

3) 21 trajectories is quite a low number for one of the data sets.

Response: We thank the reviewer's comments and have increased the trajectory number.

4) Comparing chromosome shapes to orange or lemon is not helpful and I wasn't quite sure what this meant. There are more scientific terms that can be used.

Response: We agree with the insufficient clarity of the terminology chosen for describing dynamic chromosome deformation. We have replaced the terms, orange and lemon, with chromosome deformation.

Second decision letter

MS ID#: JOCES/2022/260137

MS TITLE: Single Chromosome Dynamics Reveals Locus-Dependent Dynamics and Chromosome Territory Orientation

AUTHORS: Yu-Chieh Chung, Madhoolika Bisht, Jenna Thuma, and Li-Chun Tu
ARTICLE TYPE: Research Article

We have now reached a decision on the above manuscript.

To see the reviewers' reports and a copy of this decision letter, please go to: https://submit-jcs.biologists.org and click on the 'Manuscripts with Decisions' queue in the Author Area. (Corresponding author only has access to reviews.)

As you will see, the reviewer gave favourable reports but raised some critical points that will require amendments to your manuscript. I think that these are monir and can be completed without needing further review and I hope that you will be able to carry these out because I would like to be able to accept your paper.

Please ensure that you clearly highlight all changes made in the revised manuscript. Please avoid using 'Tracked changes' in Word files as these are lost in PDF conversion.

I should be grateful if you would also provide a point-by-point response detailing how you have dealt with the points raised by the reviewers in the 'Response to Reviewers' box. Please attend to all of the reviewers' comments. If you do not agree with any of their criticisms or suggestions please explain clearly why this is so.

Reviewer 1

Advance summary and potential significance to field

We appreciate the author's efforts to address the concerns of the reviewers. I thank the authors for their diligence in their careful responses and have only minor queries. The authors extended their current study by including data showing how the chromosome 19 q arm compaction is altered in different cell cycle stages and the effects of transcription on genomic locus mobility. The insights they derive from these experiments are potentially meaningful but I had a few clarifying questions and comments.

Comments for the author

In regards to the chromatin compaction across cell cycle data presented in Figure 2C, how do the authors show that the differences across the cell cycle are significant given the overlapping error bars? In other sections of the paper, the authors use a 1 way ANOVA test to calculate significance

© 2023. Published by The Company of Biologists under the terms of the Creative Commons Attribution License (https://creativecommons.org/licenses/by/4.0/).

for other metrics they measure but it hasn't been calculated for this dataset. Can the authors clarify how this metric was calculated and also discuss how they accounted for cell to cell variability and temporal variability in the spatial distance calculation? The authors visually show how spatial distance changes across time in Figure 3A and in supplemental movies but it is unclear how this was accounted for in the spatial distance calculation in Figure 2C. Is the same temporal window used for all cell cycles and is temporal variability similar across different cell cycles? How does cell to cell variability and temporal variability affect calculation of the scaling exponent? Can the authors show a distribution of spatial distance values similar to Figure 4C? Showing how temporal variability and cell to cell variability affects the spatial distance calculation and using the appropriate statistical significance test will provide more insight into the cell cycle affects chromatin compaction. Since the data is already available and much of the analysis has been done for other datasets in the paper, I hope that these additions aren't too burdensome.

Also, in line 52 of the abstract and in the keyword section, the authors should replace single-molecule microscopy with single particle tracking to avoid confusion since many molecules making up a single locus, not a single fluorophore is being tracked.

All other comments and questions were sufficiently addressed.

Second revision

Author response to reviewers' comments

In regards to the chromatin compaction across cell cycle data presented in Figure 2C, how do the authors show that the differences across the cell cycle are significant given the overlapping error bars?

In other sections of the paper, the authors use a 1 way ANOVA test to calculate significance for other metrics they measure but it hasn't been calculated for this dataset.

The compaction scaling exponents were obtained by fitting the power-law relation of spatial distance and genomic distance, in which several data were involved in generating one scaling exponent value. To demonstrate compactions of the chromosome are significantly different between early G1 and other cell cycle stages (late G1 and/or S), we added the significance analysis of spatial distances between pairs of loci in our revised manuscript (Fig S5). These results indicate that chromosome compaction was significantly loose in late G1 and S than the compaction in early G1 phase.

Can the authors clarify how this metric was calculated and also discuss how they accounted for cell to cell variability and temporal variability in the spatial distance calculation?

We have added the description in Materials and Methods to clarify how the compaction was calculated. The compaction curves were fitted by the power-law relation of average spatial distance and genomic distance. The average spatial distance of each pair of loci was calculated by averaging the spatial distance between the loci over 30 time frames (time average) and followed by the cell-population average. The error bars in Fig 2C are SD of the cell-population average. As shown in Fig S6, the temporal variability over time, calculated by the instantaneous deviation of spatial distance at each time point from the time average of the spatial distance (refer to the Materials and Methods for details), is mostly in the range of around 0.04 - 0.15 μm in all cell cycle stages. The cell-cell variability of the spatial distance of locus pairs (mostly around 0.5 - 2 μm) is about 10-fold higher than its temporal variability, indicating that the error bars (SD) in Fig 2C mainly come from cell-cell variability.

The authors visually show how spatial distance changes across time in Figure 3A and in supplemental movies but it is unclear how this was accounted for in the spatial distance calculation in Figure 2C. Is the same temporal window used for all cell cycles and is temporal variability similar across different cell cycles?

© 2023. Published by The Company of Biologists under the terms of the Creative Commons Attribution License (https://creativecommons.org/licenses/by/4.0/).

Fig 3A and movie S1 show typical data for locus dynamics analysis in which the movement of a genomic locus was monitored for 120 frames (~ 80 seconds). For each pair of loci in Fig 2C, the average spatial distance was calculated by averaging spatial distance over 30 frames (~19 seconds). The temporal window for all cell cycle stages was the same. As shown in Fig S6, the temporal variability across all cell cycle stages within this short time window was similar – mainly within the range between 0.04 - 0.15 µm.

How does cell to cell variability and temporal variability affect calculation of the scaling exponent? Can the authors show a distribution of spatial distance values similar to Figure 4C? Showing how temporal variability and cell to cell variability affects the spatial distance calculation and using the appropriate statistical significance test will provide more insight into the cell cycle affects chromatin compaction.

We thank the reviewer's comment. We have added the analysis of cell-to-cell variability and temporal variability in our revised manuscript. In summary, cell-to-cell variability has major contributions to the SD of cell cycle-dependent compaction calculation compared to the temporal variability. This result emphasizes the importance of live-cell experiments in which cell-cell variety can be separately analyzed during chromosome reorganization in single cells.

Since the data is already available and much of the analysis has been done for other datasets in the paper, I hope that these additions aren't too burdensome.

Also, in line 52 of the abstract and in the keyword section, the authors should replace single-molecule microscopy with single particle tracking to avoid confusion since many molecules making up a single locus, not a single fluorophore is being tracked.

We thank the reviewer's comments and have replaced single-molecule microscopy with single-particle tracking.

Third decision letter

MS ID#: JOCES/2022/260137

MS TITLE: Single Chromosome Dynamics Reveals Locus-Dependent Dynamics and Chromosome Territory Orientation

AUTHORS: Yu-Chieh Chung, Madhoolika Bisht, Jenna Thuma, and Li-Chun Tu
ARTICLE TYPE: Research Article

I am happy to tell you that your manuscript has been accepted for publication in Journal of Cell Science, pending standard ethics checks.

© 2023. Published by The Company of Biologists under the terms of the Creative Commons Attribution License (https://creativecommons.org/licenses/by/4.0/).
